# Impact of perinatal anxiety on healthcare utilisation: a matched cohort study using electronic health records in the UK

**DOI:** 10.1136/bmjopen-2025-100543

**Published:** 2026-09-25

**Authors:** Dahai Yu, James Bailey, Carolyn A Chew-Graham, Holly C Smith, Charlotte Archer, Jonathan Evans, Tamsin Fisher, David Kessler, Tom Kingstone, Irene Petersen, Janine Proctor, Noureen Shivji, Victoria Silverwood, Amy Spruce, Katrina Turner, Pensée Wu, Miliça Blagojevic-Bucknall, Kelvin P Jordan

**Affiliations:** 1 Primary Care Centre Versus Arthritis, School of Medicine, Keele University, Keele, UK; 2 Department of Primary Care and Population Health, University College London, London, UK; 3 Population Health Sciences, University of Bristol Medical School, Bristol, UK; 4 Just Family Community Interest Company (CIC), Stoke on Trent, UK

**Keywords:** Anxiety disorders, Depression & mood disorders, OBSTETRICS, Hospitalization, Electronic Health Records, Primary Health Care

## Abstract

**Abstract:**

**Objectives:**

To examine differences in healthcare utilisation patterns among women with perinatal anxiety (PNA) compared with women with anxiety but not pregnant and women with pregnancy but no anxiety.

**Design:**

A retrospective cohort study using electronic health records from the Clinical Practice Research Datalink (CPRD) Aurum (1998–2020), linked to Hospital Episode Statistics, the Index of Multiple Deprivation, Mother Baby Link and Pregnancy Register in England.

**Setting:**

Primary care records from general practices contributing to CPRD in England.

**Participants:**

The study included records for 103 287 women with PNA and two matched comparison cohorts: 413 148 women with anxiety but no pregnancy (anxiety-only cohort) and 413 148 women with pregnancy but no anxiety (pregnancy-only cohort). Women were matched 1:4 on age, general practice and index year.

**Primary and secondary outcome measures:**

Healthcare utilisation was assessed by primary care clinical encounters (recorded primary care consultations/contacts, including general practitioner (GP) and other primary care clinician contacts), prescriptions, referrals and hospitalisations. Outcomes were measured in five time periods: (1) pregnancy to delivery, (2) delivery to 72 days post partum, (3) 73 days to 6 months post partum, (4) 7–12 months post partum and (5) 13–24 months post partum. Incidence rate ratios (IRR) and absolute risk differences were calculated using conditional Poisson regression models adjusting for sociodemographic and comorbidity-related confounders.

**Results:**

Women with PNA consistently had higher rates of healthcare utilisation across all time periods. Compared with the anxiety-only cohort, the adjusted IRR for primary care encounters was 1.742 (95% CI 1.739 to 1.746) during pregnancy and 2.288 (95% CI 2.283 to 2.293) in the 72 days postpartum period. compared with the pregnancy-only cohort, the adjusted IRR for primary care encounters was 1.653 (95% CI 1.651 to 1.655) during pregnancy and 2.319 (95% CI 2.314 to 2.324) in the 72 days postpartum period. Prescriptions and referrals followed similar patterns, while increased hospitalisation rates postdelivery were not observed in the PNA cohort.

**Conclusions:**

Women with PNA exhibited higher healthcare utilisation up to 24 months after delivery, particularly in the period immediately after delivery. These findings highlight the necessity for early identification and sustained support for this population.

**STRENGTHS AND LIMITATIONS OF THIS STUDY**

- This study uses a large, nationally representative dataset of electronic health records from Clinical Practice Research Datalink Aurum, linked to Hospital Episode Statistics, the Index of Multiple Deprivation, Mother Baby Link and Pregnancy Register covering over two decades (1998–2020).
- The use of a matched cohort design enables robust comparisons between women with perinatal anxiety (PNA), women with anxiety but not pregnant and women with pregnancy but no anxiety, controlling for key confounders such as age, practice and index year.
- By examining healthcare utilisation across five distinct perinatal time periods, this study provides a detailed temporal profile of primary care encounters, prescriptions, referrals and hospitalisations in women with PNA, offering novel insights into healthcare demand.
- As with most studies based on routine healthcare data, our findings are subject to potential misclassification and incomplete recording of diagnoses and prescriptions in primary care records.
- Some important lifestyle factors, such as social support, psychological therapy use and detailed socioeconomic measures, were not available in the dataset, which may have influenced the observed associations.

## Introduction

 Perinatal anxiety (PNA) is increasingly recognised as a significant health burden for the individual and health system. A systematic review (including 26 studies, covering studies published from the inception of the database until April 2022 across global settings) estimated the prevalence of self-reported anxiety to range from 18–25% during pregnancy with prevalence of a clinical diagnosis of anxiety of 15% during pregnancy and 10% postnatal.^1^ PNA can be associated with difficulties in infant feeding, maternal substance misuse and heightened suicide risk in women, highlighting the importance of early identification and management of this problem by healthcare services.^1–3^ The prevalence and impact of PNA on women’s long-term health and infant outcomes, including developmental delays and behavioural problems,^4^ have prompted calls for better integration of mental healthcare into maternal services, supported by policies from organisations such as the WHO and the National Institute for Health and Care Excellence (NICE), particularly in settings where barriers to care exacerbate these challenges to integration.^5 6^

While previous research acknowledges increased healthcare demands among women with PNA, critical gaps remain in our understanding of the long-term trajectories of healthcare utilisation patterns beyond the immediate postnatal period.^7^ This includes a lack of detailed knowledge about how these patterns vary across primary and secondary healthcare services.^8 9^ Although the UK primary care studies have described marked changes in consultation patterns across pregnancy and the early postnatal period, with service use stabilising after the first months post partum,^7 8^ these studies did not focus on women with PNA or provide matched comparisons. Moreover, there is limited understanding of how women with PNA engage with healthcare services across different sociodemographic and ethnic groups, suggesting a need for more focused studies in this area.^9 10^

Although both anxiety and pregnancy independently incur healthcare costs and demands,^11 12^ the unique interplay and combined impact of pregnancy and anxiety on healthcare resources—particularly the differential patterns of service use and periods of greatest utilisation of healthcare services—are less understood. Such an understanding is critical, as it may reveal specific healthcare needs and intervention points that are distinctive to women with PNA. Addressing this gap could enhance healthcare systems’ and clinicians’ability to develop finely tuned healthcare policies and provision that acknowledge and cater to the nuanced differences between these groups.^13^

The current study addresses these gaps by using a large primary care dataset to determine healthcare utilisation among women living in England with PNA. Our aim is to investigate healthcare utilisation patterns, assess systemic implications, and inform healthcare interventions for women with PNA. By comparing women with PNA to matched cohorts of women experiencing anxiety without pregnancy, and pregnancy without anxiety and pregnancy without anxiety, we aimed to quantify differences in healthcare utilisation in primary care (clinical encounters, prescriptions and referrals) and secondary care (hospital admission) during pregnancy and up to 24 months post partum.

## Methods

### Setting

In the UK, primary care is typically the first point of contact for healthcare and acts as the ‘gatekeeper’ to the rest of the health service. While a newly pregnant woman can refer herself directly to a midwife service, they are likely to be seen in primary care for other health problems. Information about patients’ health is collected during consultations. While primarily used for clinical care it is also used for research through large healthcare databases.

For this study, we used the Clinical Practice Research Datalink (CPRD) Aurum,^13^ which contains anonymised medical records of over 40 million patients in the UK collected by over 1500 participating general practices.^13^ It covers general practices that use the Egton Medical Information Systems Web software, with around 20% of UK general practices contributing data^13^ including demographics, diagnoses and prescriptions. We included women with linked primary care records to area-based deprivation through the Index of Multiple Deprivation (IMD) and to Hospital Episode Statistics (HES). This linkage is available for around 75% of patients in England, whose general practices have consented to the CPRD linkage scheme. Within CPRD, the CPRD Pregnancy Register is an algorithm that takes information from maternity, antenatal and birth health records from primary care to detect pregnancies and their outcomes.^14–16^

### Study population

The study’s exposed cohort comprises women aged 15 to 49 years who experienced a live birth between 1 January 1998 and 31 December 2020, with a diagnosis of anxiety during the perinatal period. The perinatal period was defined from the start of pregnancy to 12 months post partum, with the start of pregnancy determined using the CPRD Mother Baby Link and Pregnancy Register. The diagnosis was identified through recorded anxiety diagnoses coded using Systematised Nomenclature of Medicine-Clinical Terms and/or prescriptions for anxiolytic drugs or selective serotonin reuptake inhibitors within the specified period (https://www.keele.ac.uk/hcdr/codelists/; also online supplemental tables 1 and 2). Two comparison cohorts were established. The first comparison cohort included women diagnosed with anxiety but without a pregnancy record in the same or subsequent year. The second cohort comprised women with a pregnancy record but no anxiety diagnosis recorded within the same timeframe. For each exposed woman, up to four matched unexposed cohorts from each comparison group were selected based on GP practice, age and pregnancy year. The index date for PNA women was the pregnancy start date, which was also assigned to their matched counterparts. All participants were followed from the index date until earliest date of: death, practice transfer, last collection of practice records or 31 December 2021. Each eligible participant had to have continuous registration at their general practice (with linkage to HES & IMD) for at least 2 years before and 2 years after the index date to be incorporated into matched cohorts.

### Outcomes

The primary outcomes for this study were healthcare utilisation measured by the number of primary care clinical encounters (recorded primary care consultations/contacts in primary care records, including GP and other primary care contacts), the number of different medications prescribed, the total number of primary care referral records to specialist care/secondary care (Referral attendance was not assessed and referrals were analysed as a separate utilisation outcome, not as a primary care clinical encounter), and the total number of hospital admissions over the follow-up period. Specific follow-up timeframes for these measurements were from the start of pregnancy to delivery (period 1), 1 day postdelivery to 72 days post partum (period 2), 73 days to 6 months post partum (period 3), 7 to 12 months post partum (period 4) and 13 to 24 months post partum (period 5). A cut-off of 72 days post partum was used based on prior evidence suggesting that healthcare consultation rates, particularly for postnatal checks and other clinical events, stabilise around this time frame after an initial peak, as shown in previous studies of maternal healthcare utilisation patterns.^17 18^

### Covariates

Covariates included age, index year, geographical region, deprivation, ethnicity, smoking status, body mass index (BMI) and comorbidities. Age at index date was presented both as a continuous variable and as a categorical variable divided into four groups: <25 years, 25–29 years, 30–34 years and ≥35 years. Index year (year of pregnancy start) was categorised into five periods: 1998–2000, 2001–2005, 2006–2010, 2011–2015 and 2016–2020, to account for period effect. Geographical region covered North East, North West, Yorkshire and The Humber, East Midlands, West Midlands, East of England, London, South East, South West and Northern Ireland, and was included as a covariate to control for regional variations in healthcare access and delivery. Neighbourhood deprivation was categorised based on the IMD (an area-level measure of relative socioeconomic deprivation in England). This is a composing measure covering several domains (health deprivation and disability; barriers to housing and services; employment; income; education, skills and training; crime; living environment). IMD was categorised into deciles (IMD 1=least deprived to IMD 10=most deprived), with an additional category for those without IMD linkage. Ethnicity was derived from the HES linkage and categorised into six groups: white, Black, Asian, mixed, other and unknown. Based on the latest information recorded within 24 months prior to the index date, smoking status was classified as current smoker versus non/ex-smoker. BMI was categorised as a binary variable for obesity (BMI ≥30 kg/m^2^) based on the latest records within 24 months prior to index date. Comorbidity status was dichotomised as no comorbidity (0) vs comorbidity (≥1) based on Charlson comorbidity index, which summarises pre-existing chronic physical conditions recorded within 24 months prior to index date and does not represent psychological comorbidity.

### Statistical analysis

Continuous covariates are presented as mean (SD), and categorical variables are presented as count (percentage). Our analyses focused on population-level healthcare utilisation (rates of events per person-time) within predefined perinatal periods, rather than individual-level longitudinal trajectory modelling. Absolute rates of outcomes (per 1000 person-years) of each outcome were estimated for each of the follow-up time periods using Poisson regression models. By partitioning follow-up into five predefined perinatal periods, we compared period-specific utilisation rates and associations to describe how differences between cohorts vary across pregnancy and post partum windows. To estimate the association between PNA and rate of outcomes, conditional Poisson regression models were fitted unadjusted and adjusted for ethnicity, geographical region, deprivation, comorbidity, obesity and smoking status. Both relative associations (incidence rate ratio ((IRR)) and absolute associations (absolute risk difference (ARD)) with 95% CIs were estimated. Because we modelled rates within non-overlapping periods and inference is based on between-cohort contrasts within each period, our conclusions are not dependent on modelling within-person random effects.

Sensitivity analyses were applied to test the consistency of the estimations derived from the main analyses. Adjusted IRRs for each outcome were estimated in each calendar index year to examine the period effect. Missing data on ethnicity, IMD, smoking status and obesity status in the main analysis were recorded in a ‘missing’ category but sensitivity analyses were undertaken to compare the estimations using data with a missing category with estimations from the complete case analysis.

Because women contribute observations across multiple perinatal periods (and some contribute more than one pregnancy episode), we conducted sensitivity analyses to account for within-person correlation across repeated period-specific observations. We restructured the dataset into long format with one record per woman–episode–period and refitted models using (1) Poisson regression with robust SEs clustered at the woman level and (2) population-averaged Poisson GEE models (log link, Poisson family) with an exchangeable working correlation and robust variance. Period-specific IRRs were derived from cohort-by-period interaction terms. All analyses were performed using Stata/MP V.18.0 (StataCorp).

### Patient and public involvement

Patient and public involvement (PPI) was integral to the design and development of this study through engagement with the CLAssification and outcomeS of Perinatal anxiety’s Patient Advisory and Clinical Advisory Groups. Feedback was sought on all study objectives, including the database study reported in this paper, particularly regarding the relevance of examining healthcare utilisation patterns in women PNA. The groups emphasised the importance of identifying disparities in access to care and the need for clear comparisons between different patient groups. Based on this input, we refined our approach to highlight differences in healthcare utilisation across multiple time windows post partum. Our advisory groups helped shape the study’s focus, interpretation of findings and how to disseminate our findings to different audiences. PPI input was advisory and did not involve collection of qualitative research data (eg, interviews), therefore no quotations or qualitative analytic tools apply.

## Results

In total, 103 287 women with PNA, 413 148 women with anxiety but no pregnancy, and 413 148 pregnant women but no anxiety were included in the study. The mean age was 29.8 years (SD 5.9) across all cohorts (table 1).

**Table 1:** Baseline characteristics of women in the PNA and matched cohorts

	PNA cohort	Matched cohort 1	Matched cohort 2
	Perinatal anxiety	Anxiety without pregnancy	Pregnancy without anxiety
n	103 287	413 148	413 148
Age, mean (SD)	29.8 (5.9)	29.8 (5.9)	29.8 (5.9)
Age group, n (%)			
<25 years	22 124 (20.5)	88 496 (20.5)	88 496 (20.5)
25–29 years	28 160 (26.1)	112 640 (26.1)	112 640 (26.1)
30–34 years	32 638 (30.3)	130 552 (30.3)	130 552 (30.3)
≥35 years	24 953 (23.1)	99 812 (23.1)	99 812 (23.1)
Index Year, n (%)			
1998–2000	10 500 (10.2)	42 000 (10.2)	42 000 (10.2)
2001–2005	21 948 (21.3)	87 792 (21.3)	87 792 (21.3)
2006–2010	19 051 (18.4)	76 204 (18.4)	76 204 (18.4)
2011–2015	28 629 (27.7)	114 516 (27.7)	114 516 (27.7)
2016–2020	23 159 (22.4)	92 636 (22.4)	92 636 (22.4)
Geographical region, n (%)			
North East	4440 (4.1)	17 760 (4.1)	17 760 (4.1)
North West	23 040 (21.4)	92 160 (21.4)	92 160 (21.4)
Yorkshire and The Humber	4127 (3.8)	16 508 (3.8)	16 508 (3.8)
East Midlands	2527 (2.3)	10 108 (2.3)	10 108 (2.3)
West Midlands	15 766 (14.6)	63 064 (14.6)	63 064 (14.6)
East of England	4980 (4.6)	19 920 (4.6)	19 920 (4.6)
London	11 680 (10.8)	46 720 (10.8)	46 720 (10.8)
South East	26 948 (25.0)	107 792 (25.0)	107 792 (25.0)
South West	13 408 (12.4)	53 632 (12.4)	53 632 (12.4)
Northern Ireland	959 (0.9)	3836 (0.9)	3836 (0.9)
Decile of Index of Multiple Deprivation, n (%)			
Least deprivation	10 478 (9.7)	37 659 (8.7)	42 265 (9.8)
IMD=2	9657 (9.0)	37 752 (8.8)	38 589 (8.9)
IMD=3	9890 (9.2)	39 579 (9.2)	40 224 (9.3)
IMD=4	9998 (9.3)	40 587 (9.4)	39 791 (9.2)
IMD=5	9224 (8.6)	38 060 (8.8)	36 827 (8.5)
IMD=6	9939 (9.2)	41 503 (9.6)	39 475 (9.2)
IMD=7	10 637 (9.9)	44 649 (10.4)	40 995 (9.5)
IMD=8	10 689 (9.9)	43 768 (10.1)	40 066 (9.3)
IMD=9	11 744 (10.9)	47 618 (11.0)	43 837 (10.2)
Most deprivation	13 428 (12.5)	51 495 (11.9)	50 872 (11.8)
Without IMD linkage	2191 (2.0)	8830 (2.1)	18 559 (4.3)
Ethnicity group, n (%)			
White	92 968 (86.2)	309 378 (71.7)	326 395 (75.6)
Black	2007 (1.9)	4849 (1.1)	14 117 (3.3)
Asian	3435 (3.2)	8041 (1.9)	24 311 (5.6)
Mixed	1102 (1.0)	3029 (0.7)	4905 (1.1)
Other	1696 (1.6)	4581 (1.1)	9572 (2.2)
Unknown	6667 (6.2)	101 622 (23.6)	52 140 (12.1)
Recorded current smoker, n (%)	25 682 (23.8)	71 514 (16.6)	74 628 (17.3)
Recorded BMI, n (%)	47 359 (45.9)	200 337 (48.5)	169 440 (41.0)
BMI, mean (SD), kg/m^2^	26.7 (6.4)	27.5 (7.7)	25.9 (6.0)
Recorded obesity, n (%)	11 980 (25.0)	61 081 (30.0)	35 283 (21.0)
≥1 Charlson comorbidities, n (%)	13 423 (12.4)	67 349 (15.6)	41 058 (9.5)

Column headings show the number of unique women. A woman could contribute to more than one matched risk set, so counts summarised across risk-set inclusions may exceed the number of unique women.

BMI, body mass index ; PNA, perinatal anxiety.

Rates of each healthcare utilisation outcome over all periods are presented in table 2, with adjusted IRRs shown in online supplemental table 3. The population with PNA were more likely to have higher rates of outcomes during most periods. The main exception was for hospitalisations after 73 days from delivery.

**Table 2:** Outcome rates per 1000 person-years (95% CI)

	PNA cohort	Matched cohort 1	Matched cohort 2
	Perinatal anxiety	Anxiety without pregnancy	Pregnancy without anxiety
Period 1: Index date-delivery date			
Primary care clinical encounters	18 356.6 (18 331.1 to 18 382.2)	9947.6 (9938.1 to 9957.0)	10 681.6 (10 670.8 to 10 690.3)
Referrals	39.3 (38.2 to 40.5)	61.0 (60.3 to 61.8)	13.3 (13.0 13.7)
Prescriptions	3351.1 (3340.2 to 3362.1)	2605.2 (2600.4 to 2610.1)	2004.6 (2000.3 to 2008.8)
Hospitalisations	1381.4 (1374.4 to 1388.5)	236.4 (234.9 to 237.8)	788.3 (785.6 to 790.9)
Period 2: delivery date-72 days post partum			
Primary care clinical encounters	6740.0 (6724.5 to 6755.5)	2815.5 (2810.5 to 2820.5)	2815.5 (2810.5 to 2820.5)
Referrals	19.2 (18.4 to 20.0)	12.9 (12.5 to 13.2)	3.8 (3.6 to 4.0)
Prescriptions	2049.6 (2041.0 to 2058.1)	1188.1 (1184.8 to 1191.3)	741.5 (738.9 to 744.0)
Hospitalisations	186.2 (183.7 to 188.8)	60.6 (59.8 to 61.3)	218.3 (217.0 to 219.7)
Period 3: 73 days-6 months post partum			
Primary care clinical encounters	5482.4 (5468.5 to 5496.4)	4106.1 (4100.0 to 4112.1)	3763.6 (3757.8 to 3769.4)
Referrals	27.6 (26.6 to 28.6)	18.1 (17.7 to 18.5)	5.5 (5.3 to 5.7)
Prescriptions	1881.7 (1873.5 to 1889.9)	1476.7 (1473.1 to 1480.4)	983.9 (980.9 to 986.8)
Hospitalisations	75.8 (74.2 to 77.5)	93.3 (92.4 to 94.2)	267.2 (265.6 to 268.7)
Period 4: 7–12 months post partum			
Primary care clinical encounters	8400.3 (8383.0 to 8417.6)	6437.1 (6429.5 to 6444.7)	5287.1 (5280.2 to 5293.9)
Referrals	40.6 (39.5 to 41.9)	29.3 (28.8 to 29.8)	9.1 (8.8 to 9.4)
Prescriptions	2303.7 (2294.7 to 2312.8)	1528.3 (1524.6 to 1532.0)	981.5 (978.5 to 984.4)
Hospitalisations	138.5 (136.3 to 140.8)	155.7 (154.4 to 156.8)	275.2 (273.6 to 276.8)
Period 5: 13–24 months post partum			
Primary care clinical encounters	15 277.0 (15 253.7 to 15 300.4)	11 696.2 (11 686.0 to 11 706.5)	9360.7 (9351.6 to 9369.9)
Referrals	50.6 (49.3 to 52.0)	54.2 (53.5 to 54.9)	17.3 (16.9 to 17.7)
Prescriptions	2996.9 (2986.6 to 3007.2)	2073.4 (2069.1 to 2077.7)	1449.0 (1445.4 to 1452.6)
Hospitalisations	340.7 (337.2 to 344.2)	309.5 (307.9 to 311.2)	373.0 (371.2 to 374.9)

PNA, perinatal anxiety.

### Period 1 (index date to delivery)

During period 1 (index date to delivery), primary care clinical encounter rates in the PNA cohort were 60%–70% higher than those in the matched cohorts (adjusted IRRs: compared with cohort with anxiety but no pregnancy 1.742 (95% CI 1.739 to 1.746), compared with cohort with pregnancy but no anxiety 1.653 (95% CI 1.651 to 1.655)) (online supplemental table 3). Prescription and hospitalisation rates were also higher in the PNA cohort than the matched cohorts, with hospitalisation rates more than five times higher than in the cohort with anxiety but no pregnancy (IRR 5.155 (95% CI 5.128 to 5.182)). Referral rates were nearly three times higher in the PNA cohort compared with the cohort with pregnancy but no anxiety (IRR 2.841 (95% CI 2.725 to 2.949)) but lower than the cohort with anxiety but no pregnancy (IRR 0.600 (95% CI 0.581 to 0.621)). Period 2 (delivery to 72 days post partum): The biggest difference in primary care encounters was seen in period 2 (delivery to 72 days post partum), with the PNA cohort having more than double the rate of encounters as the cohort with anxiety but no pregnancy (IRR 2.288 (95% CI 2.283 to 2.293)) and the cohort with pregnancy but no anxiety (IRR 2.319 (95% CI 2.314 to 2.324)). Prescription and referral rates were also higher in the PNA cohort during this period compared with both matched cohorts, with the bigger difference being in comparison to the pregnancy-only cohort. Hospitalisation rates were higher in the PNA cohort compared with the cohort with anxiety but no pregnancy, but lower than in the cohort with pregnancy but no anxiety.

### Periods 3–5 (73 days to 24 months post partum)

In period 3 (73 days to 6 months post partum), period 4 (7–12 months post partum) and period 5 (13–24 months post partum), primary care encounter, prescription and referral rates remained higher for the PNA cohort than the matched cohorts, although the difference in rates was slightly lower than for period 2. In contrast, hospitalisation rates were lower for the PNA cohorts than both matched cohorts across periods 3–5 (online supplemental table 3). The corresponding ARDs are presented in online supplemental table 4.

The strength of associations between healthcare utilisation and PNA remained relatively stable or reduced slightly across the index years. For primary care clinical encounters, the IRR in both matched cohorts remained above 1, indicating higher utilisation in the PNA cohort, though the difference has reduced in recent years (online supplemental figure 1). For referrals and prescriptions, the associations remained stable over the calendar years, with some reduction in strength in more recent years compared with the cohort with pregnancy but no anxiety in later postpartum periods (online supplemental figure 2 and 3). Associations with hospitalisations also remained stable (online supplemental figure 4).

Complete-case analyses produced estimates consistent with the primary analysis (online supplemental table 5). For example, in period 2 (delivery to 72 days post partum), the adjusted IRR for primary care clinical encounters comparing women with PNA with the anxiety-only cohort was 2.29 (95% CI 2.28 to 2.30) (main analysis: 2.29 (2.28 to 2.29)) and 2.32 (2.31 to 2.33) compared with the pregnancy-only cohort (main analysis: 2.32 (2.31 to 2.32)). Similar consistency was observed across outcomes and time windows.

Sensitivity analysis using complete cases only gave similar findings as the main analysis.

Sensitivity analyses that accounted for within-person correlation using woman-clustered robust Poisson models and population-averaged Poisson GEE models produced consistent period-specific estimates for consultation rates; Generalized estimating equations (GEE) estimates were slightly attenuated towards the null, but the direction and overall time-pattern of differences between cohorts were unchanged (online supplemental table 6).

## Discussion

This study provides a comprehensive analysis of healthcare utilisation patterns in women with PNA compared with matched cohorts of women experiencing only anxiety or only pregnancy, using a large dataset of primary care records. Our findings show that women with PNA have consistently higher rates of primary care clinical encounters, prescriptions and referrals to specialist care up to 24 months after delivery, indicating that healthcare demands of women with PNA are significantly higher. These findings are consistent with evidence that perinatal mental health problems are associated with increased healthcare use and costs, although much of the existing literature has focused on perinatal depression or mixed perinatal mental health populations rather than anxiety-specific cohorts.^12 13 19 20^ UK primary care studies have also demonstrated marked changes in consultation patterns around childbirth, particularly in the early postnatal period, but have not provided matched comparisons focused on PNA.^18 21^

This study presents several novel findings of healthcare utilisation among women with PNA. It offers a detailed temporal analysis over two decades, showing that healthcare utilisation rates have been consistently higher in the PNA cohort. By comparing PNA to both anxiety-only and pregnancy-only cohorts, the study highlights unique healthcare needs specific to PNA. Notably, while hospitalisation rates were also elevated prior to the end of pregnancy in the PNA cohort, these differences decreased in the later postpartum periods.^21^ Given also that the elevated rates of primary care encounters, referrals and prescriptions were highest during pregnancy and in the initial period post partum, this study highlights the critical need for targeted healthcare interventions to identify and support women with PNA, particularly between the start of pregnancy and the first 72 days post partum.

The findings of this study extend previous research indicating that PNA is associated with increased healthcare utilisation. Where Humphrey *et al*^22^ underscore the critical need for comprehensive maternal healthcare due to risks associated with maternal morbidity and mortality, our study highlights increased healthcare demands in women with PNA. The prevalence and risk factors identified by Giardinelli *et al* are consistent with our observation of greater primary care encounters and prescription rates in the PNA cohort.^23^ Both studies highlight the need for early identification and intervention to mitigate the adverse effects of PNA. Ding *et al*^24^ linked maternal anxiety to adverse birth outcomes such as preterm birth, low birth weight and small-for-gestational-age babies. These adverse outcomes may contribute to the increased healthcare use post partum we found in our study. By providing detailed temporal patterns of healthcare utilisation, our study adds to this body of evidence, demonstrating persistent elevated utilisation even as some differences diminish over time.

The findings from this study have significant implications for maternal mental and perinatal healthcare policy and practice. The consistently higher rates of healthcare utilisation among women with PNA, especially the increased primary care encounters and prescription rates, underscore the necessity for comprehensive, integrated services across primary, maternal and mental healthcare settings. These results suggest that healthcare systems should prioritise early identification and proactive support of women with PNA to address their unique and evolving healthcare needs, as early intervention and support can reduce long-term healthcare costs and improve outcomes for both mother and their children.^11 25^ By understanding the specific needs and risks associated with PNA, commissioners and practitioners should prioritise the provision of tailored interventions that address both mental and physical health, potentially reducing the overall healthcare burden and improving outcomes for this vulnerable population. These findings support the integration of mental health screening and support into routine prenatal and postnatal care, as recommended by the NICE.^23^

The identification of a critical intervention window between pregnancy and delivery, and in the first few weeks after delivery, where healthcare utilisation rates were particularly high in the PNA cohort compared with both the anxiety-only and pregnancy-only cohorts, highlights the need for targeted support during this period. The higher rates of hospitalisation in the PNA group in these periods compared with the pregnancy-only cohort may reflect additional healthcare needs associated with PNA above that seen during pregnancy. This aligns with recommendations including from the Royal College of Obstetricians and Gynaecologists,^26^ which emphasise the importance of post partum mental healthcare, particularly the maternal post-natal check, conducted in primary care, to prevent long-term adverse outcomes for both mothers and their children.^24 27^

This study has notable strengths. First, the large sample size and longitudinal data, derived from a national database of anonymised primary care records, provide robust statistical power and generalisability. The inclusion of over 103 000 women with PNA and matched unexposed cohorts totalling over 826 000 individuals ensures a comprehensive analysis that reflects real-world scenarios. One of the key strengths is the utilisation of a CPRD-derived algorithm to obtain improved details on pregnancy. This methodological approach improves the accuracy and validity of case identification, reducing misclassification bias.^28^ The study design also includes carefully matched unexposed cohorts based on pregnancy date, practice and age, which improves statistical efficiency and controls for potential confounding variables. By including both an anxiety-only matched unexposed group and a pregnancy-only matched unexposed group, the study can isolate the unique effects of PNA on healthcare utilisation, providing clearer insights into the distinct healthcare needs of this population. Furthermore, the longitudinal nature of the data allows for the examination of period effects, showing how the association between PNA and healthcare outcomes evolves over time. This analysis is crucial for understanding the long-term impact of PNA and identifying critical periods for intervention

There are several limitations that should be acknowledged. First, there might still be unmeasured confounders that were not accounted for, potentially influencing the observed associations. Another limitation is the potential for misclassification or underdiagnosis of PNA in the pregnancy cohort. Some cases of PNA may not have been diagnosed or recorded during pregnancy, leading to an underestimation of the true prevalence and impact of PNA on healthcare utilisation. Not all women with PNA will seek help and delayed diagnoses and treatments might result in an underrepresentation of the actual burden of PNA in the studied cohort. The reliance on recorded diagnoses and prescriptions may result in missing cases where anxiety is managed without formal diagnostic coding or medication. Women with less severe or subclinical anxiety symptoms^20^ might not receive recorded interventions or formal care, leading to an underrepresentation of their healthcare utilisation in the data. This under-reporting could bias the findings by primarily capturing individuals with more severe or formally diagnosed conditions. In addition, use of psychological therapies (eg, talking therapies/improving access to psychological therapies) is not comprehensively recorded in primary care; therefore, we could not adjust for receipt of non-pharmacological treatment. Residual confounding may remain because some important determinants of healthcare utilisation (eg, detailed obstetric history captured in maternity systems, healthcare access factors, prior utilisation patterns, and more granular medication use) were not available within the linked datasets used in this study. Future studies with access to richer data sources should consider incorporating these domains and using more systematic covariate selection strategies to further strengthen inference.

## Conclusions

The findings underscore the critical need for prompt detection and proactive support for women experiencing PNA. Implementing early screening and providing appropriate interventions can alleviate the adverse impacts associated with PNA, thereby enhancing health outcomes for both mothers and their infants. Additionally, exploring the underlying mechanisms of PNA and developing targeted interventions are crucial steps to enhance healthcare delivery and outcomes for this vulnerable group. These efforts will contribute to a more comprehensive understanding and management of women with PNA, ultimately benefiting both mothers and their children.

## Supplementary material

10.1136/bmjopen-2025-100543 online supplemental file 1

## Data Availability

Data may be obtained from a third party and are not publicly available.
