# Supplementary material for: Impact of perinatal anxiety on healthcare utilisation: a matched cohort study using electronic health records in the UK
Source: BMJ Open. 2026 Sep 25;16(9):e100543. doi: 10.1136/bmjopen-2025-100543 (PMC13629954; doi:10.1136/bmjopen-2025-100543)
Supplement: Supplementary file 1 [file bmjopen-16-9-s001.pdf]

Supplemental Table 1. SNOMED codes used to define PNA.

medcodeid	OriginalReadCode	CleansedReadCode	Term	SnomedCTConceptId	SnomedCTDescriptionId
252973015	1B1Z	1B1Z.00	general nervous symptom nos	424196004	2643154017
253620013	2258	2258.00	o/e - anxious	162723006	253620013
294953016	E2000	E200000	anxiety state unspecified	198288003	304838011
294960010	E2004	E200400	chronic anxiety	191708009	294960010
294961014	E2005	E200500	recurrent anxiety	191709001	294961014
294963012	E200z	E200z00	anxiety state	198288003	304838011
294992011	E2021	E202100	agoraphobia with panic attacks	191722009	294992011
294993018	E2022	E202200	agoraphobia without history of panic disorder	61569007	102288011
295477014	E283	E283.00	other acute stress reactions	67195008	500648010
295478016	E2830	E283000	acute situational disturbance	192041001	295478016
295483012	E283z	E283z00	other acute stress reaction nos	67195008	500648010
296238018	Eu41	Eu41.00	[x]other anxiety disorders	197480006	303689015
296239014	Eu410	Eu41000	[x]panic disorder [episodic paroxysmal anxiety]	371631005	1210253015
296245018	Eu413	Eu41300	[x]other mixed anxiety disorders	231504006	346979010
296249012	Eu41z	Eu41z00	[x]anxiety disorder, unspecified	197480006	303689015
296271019	Eu43y	Eu43y00	[x]other reactions to severe stress	271952001	406972015
296272014	Eu43z	Eu43z00	[x]reaction to severe stress, unspecified	271952001	406972015
304838011	E200-99	E200.99	anxiety state	198288003	882361000006113
317958013	R2y2	R2y2.00	[d]nervousness	424196004	2643154017
318033015	Ryu58	Ryu5800	[x]state of emotional shock and stress, unspecified	442347009	2821158018
338135018	1B1T	1B1T.00	feeling stressed	224974006	338135018
339044013	E2001-1	E200111	panic attack	225624000	339044013
342665019	8G94	8G94.00	anxiety management training	228560001	342665019
401810015	E28z	E28z.00	acute stress reaction nos	67195008	500648010
401881014	Eu41y	Eu41y00	[x]other specified anxiety disorders	197480006	303689015
460751013	ZV4By	ZV4By00	occupation-related stress disorder	10586006	18397010
461440017	ZVu41	ZVu4100	[x]other physical and mental strain related to work	10586006	18397010
481154010	E2002	E200200	generalised anxiety disorder	21897009	481154010
500650019	E28	E28..00	acute stress reaction	67195008	500648010
504002010	1BK	1BK..00	worried	79015004	504002010
1210253015	E2001	E200100	panic disorder	371631005	1210253015
1222552017	R2y2-2	R2y2.12	[d]nervous tension	425131000	2643155016
1222553010	R2y2-1	R2y2.11	feeling nervous	424196004	2643154017
1230451012	E2924	E292400	adjustment reaction with anxious mood	47372000	1230451012
1488717011	1B1V	1B1V.00	c/o - panic attack	395017009	1488717011
1780506015	388N	388N.00	had scale: anxiety score	401319005	1780506015
2536376012	1B13	1B13.00	anxiousness	48694002	2536376012
2549138014	388b	388b.00	depression anxiety stress scales anxiety score	416619008	2549138014
2549895012	225J	225J.00	o/e - panic attack	417676004	2549895012
2882975016	38GQ	38GQ.00	short health anxiety inventory	447278000	2882975016
109281000006110	1B12-2	1B12.12	nervous tension	425131000	2643155016
138401000006119	E202-1	E202.11	social phobic disorders	25501002	42759014
291881000006114	1B12-1	1B12.11	nerves	424196004	3013701010
291891000006112	1B12	1B12.00	nervousness	424196004	3013701010
300681000000118	8HHp	8HHp.00	referral for guided self-help for anxiety	199101000000102	300681000000118
302741000006114	R00zW	R00zW00	emotional stress	442347009	2821158018
362301000006112	Eu430-1	Eu43011	[x]acute crisis reaction	67195008	500647017
362401000006115	Eu430-2	Eu43012	[x]acute reaction to stress	67195008	500648010
362441000006118	Eu430	Eu43000	[x]acute stress reaction	67195008	500648010
362871000006111	Eu400	Eu40000	agoraphobia	70691001	117416017
362881000006114	Eu400-1	Eu40011	[x]agoraphobia without history of panic disorder	70691001	117416017
363361000006117	Eu401-1	Eu40111	[x]anthropophobia	25501002	42759014
363641000006114	Eu41y-1	Eu41y11	[x]anxiety hysteria	197480006	303689015
363651000006111	Eu411-1	Eu41111	anxiety neurosis	207363009	317740010
363661000006113	Eu41z-1	Eu41z11	[x]anxiety nos	197480006	303689015
363671000006118	Eu411-2	Eu41112	[x]anxiety reaction	197480006	303689015

363681000006115	Eu411-3	Eu41113	[x]anxiety state	198288003	304838011
375971000006116	Eu430-4	Eu43014	[x]crisis state	67195008	500648010
388071000006116	Eu411	Eu41100	[x]generalized anxiety disorder	21897009	481154010
398351000006110	Eu412-1	Eu41211	[x]mild anxiety depression	231504006	346979010
398561000006117	Eu412	Eu41200	[x]mixed anxiety and depressive disorder	231504006	346979010
403931000006116	Eu054	Eu05400	organic anxiety disorder	17496003	29572018
418031000006113	Eu410-1	Eu41011	[x]panic attack	371631005	1210253015
418051000006118	Eu400-2	Eu40012	[x]panic disorder with agoraphobia	191722009	294992011
418061000006116	Eu410-2	Eu41012	[x]panic state	371631005	1210253015
419841000006116	Eu341-4	Eu34114	[x]persistant anxiety depression	231504006	346979010
420641000006116	Eu931	Eu93100	[x]phobic anxiety disorder of childhood	192611004	296723015
423021000006114	Eu431	Eu43100	post-traumatic stress disorder	47505003	494851018
423811000006112	Eu430-5	Eu43015	[x]psychic shock	67195008	500648010
424471000006119	Eu43	Eu43.00	stress and adjustment reaction	271952001	406972015
427061000006110	Eu401-2	Eu40112	[x]social neurosis	25501002	42759014
427071000006115	Eu401	Eu40100	social phobia	25501002	42759014
429421000006116	Eu431-1	Eu43111	[x]traumatic neurosis	47505003	494848013
488201000006114	E200	E200.00	anxiety disorder	197480006	303689015
488211000006112	E2003	E200300	mixed anxiety and depressive disorder	231504006	346979010
488251000006113	1B13-1	1B13.11	anxiousness - symptom	48694002	2536376012
516601000000113	1B13-2	1B13.12	anxious	48694002	516601000000113
566931000000114	388w	388w.00	generalised anxiety disorder 7 item score	445455005	2872229011
566981000000113	388w-1	388w.11	gad-7 score	445455005	2872229011
882381000006115	E2021-99	E202199	agoraphobia	191722009	882381000006115
1808521000006110	EMISNQPA155		panic disorder without agoraphobia	56576003	94086016
1901341000006113	38QN	38QN.00	gad-2 (generalised anxiety disorder 2) scale score	836571000000106	2172211000000113
2287071000000115	173f	173f.00	anxiety about breathlessness	702535006	3004280013
2335491000000117	6897	6897.00	anxiety screening	908501000000101	2335491000000117
2335531000000117	68970	6897000	anxiety screening using questions	908521000000105	2335531000000117
2335571000000115	8CAZ0	8CAZ000	patient given advice about management of anxiety	908541000000103	2335571000000115
2404011000000118	8IH8	8IH8.00	gad-7 (generalized anxiety disorder 7) scale declined	715508000	3302874013
2668441000006114	^ESCTWO266844		work-related stress disorder	10586006	18400018
2848291000006116	^ESCTGE284829		generalized anxiety disorder	21897009	36731013
2848301000006115	^ESCTGA284830		gad - generalized anxiety disorder	21897009	481155011
2848311000006117	^ESCTGA284831		gad - generalised anxiety disorder	21897009	481156012
2907991000006110	^ESCTSO290799		social anxiety disorder	25501002	42761017
3265811000006112	^ESCTAD326581		adjustment disorder with anxious mood	47372000	78975018
3265831000006118	^ESCTAD326583		adjustment disorder with anxiety	47372000	3013688013
3268061000006113	^ESCTPO326806		posttraumatic stress disorder	47505003	79193014
3268081000006115	^ESCTPT326808		ptsd - post-traumatic stress disorder	47505003	494849017
3268091000006117	^ESCTPO326809		post-traumatic stress syndrome	47505003	494850017
3287741000006111	^ESCTAN328774		anxiety	48694002	81133019
3287761000006110	^ESCTFE328776		feeling anxious	48694002	2536377015
3287771000006115	^ESCTAN328777		anxiety reaction	48694002	2536378013
3592651000006112	^ESCTAC359265		acute stress disorder	67195008	111640012
3649691000006114	^ESCTPH364969		phobia of going out	70691001	1233303016
3785091000006118	^ESCTWO378509		worry	79015004	131118010
3785101000006112	^ESCTWO378510		worrying	79015004	504000019
3785111000006110	^ESCTAN378511		anxious cognitions	79015004	504001015
4391041000006110	^ESCTSE439104		separation anxiety	126943008	231012
4767501000006113	^ESCTCH476750		childhood phobic anxiety disorder	192611004	296723015
4934751000006114	^ESCTST493475		stressed out	224974006	338136017
4937701000006118	^ESCTAC493770		acknowledging anxiety	225216003	338442012
4944401000006116	^ESCTAN494440		anxiety about treatment	225635005	339057017
4944421000006114	^ESCTAN494442		anxiety about loss of control	225637002	339059019
4944431000006112	^ESCTAN494443		anxiety about resuming sexual relations	225638007	339060012
4944481000006113	^ESCTAN494448		anxiety about not coping with parenthood	225642005	339065019
4944501000006115	^ESCTAN494450		anxiety about making mistakes	225643000	339068017

4944521000006113	^ESCTAN494452		anxiety about altered body image	225644006	339069013
4948981000006112	^ESCTAL494898		alleviating anxiety	225977007	339505012
5024071000006118	^ESCTAN502407		anxiety depression	231504006	346980013
5024101000006111	^ESCTAN502410		anxiety hysteria	231506008	346983010
5248991000006117	^ESCTAN524899		anxiety and fear	247805009	369987018
5249061000006116	^ESCTAN524906		anxiety about body function or health	247808006	369994015
5249071000006111	^ESCTAN524907		anxiety about health	247808006	369995019
5249261000006112	^ESCTAN524926		anxiety about behaviour or performance	247825008	370017015
5249271000006117	^ESCTAN524927		anxiety about social functioning	247825008	370015011
5548991000006110	^ESCTCL554899		clinical anxiety scale	273366006	408731016
5605191000006116	^ESCTAN560519		anxiety about losing control of bowels	277818007	414533015
5605201000006118	^ESCTAN560520		anxiety about wetting self	277819004	414534014
5605231000006114	^ESCTAN560523		anxiety about choking	277822002	414537019
5605241000006116	^ESCTAN560524		anxiety about swallowing	277823007	414538012
5605251000006119	^ESCTAN560525		anxiety about collapsing	277824001	414539016
5605261000006117	^ESCTAN560526		anxiety about shaking	277825000	414540019
5605271000006112	^ESCTAN560527		anxiety about sweating	277826004	414541015
5605281000006110	^ESCTAN560528		anxiety about dying	277827008	414542010
5605341000006111	^ESCTAN560534		anxiety about becoming fat	277831002	414548014
5605371000006115	^ESCTAN560537		anxiety about fainting	277833004	414551019
5605381000006117	^ESCTAN560538		anxiety about having a heart attack	277834005	414552014
5605421000006110	^ESCTAN560542		anxiety about saying the wrong thing	277839000	414557015
5628441000006118	^ESCTPE562844		performance anxiety	279622009	416975011
5717641000006118	^ESCTLE571764		level of anxiety	286644009	426044018
5718761000006110	^ESCTCH571876		character trait finding of level of anxiety	286709003	426153011
5886461000006119	^ESCTPA588646		parental anxiety	300894000	442084019
5886481000006112	^ESCTAN588648		anxiety attack	300895004	442086017
5932991000006115	^ESCTBE593299		beck anxiety inventory	304711006	447082010
6025521000006113	^ESCTAN602552		anxiety counselling	313087008	456917011
6025551000006116	^ESCTCO602555		counselling for anxiety	313087008	2695968012
6358151000006115	^ESCTEP635815		episodic paroxysmal anxiety disorder	371631005	1229089012
6550101000006115	^ESCTCO655010		complaining of panic attack	395017009	2672971015
6651471000006117	^ESCTHO665147		hospital anxiety and depression scale: anxiety score	401319005	2972824010
7308431000006110	^ESCTGE730843		generalized anxiety disorder 7 item score	445455005	2872230018
7308441000006117	^ESCTGA730844		gad (generalised anxiety disorder) 7 item score	445455005	1666611000000117
7310811000006117	^ESCTAS731081		assessment using generalised anxiety disorder 7 item score	445598007	2871700011
7310821000006113	^ESCTAS731082		assessment using generalized anxiety disorder 7 item score	445598007	2871701010
7330171000006111	^ESCTHE733017		health anxiety inventory short week score	446793008	2884010016
7663271000006111	^ESCTMA766327		management of anxiety	710060004	3043186017
7733361000006112	^ESCTGA773336		gad-7 (generalised anxiety disorder 7) scale declined	715508000	3302875014
7733381000006119	^ESCTAS773338		assessment using generalised anxiety disorder 7 item scale declined	715508000	3302873019
8113001000006117	^ESCTST811300		stranger anxiety	351861000000106	660531000000114
8278491000006117	^ESCTAS827849		assessment using gad-2 (generalised anxiety disorder 2) scale	836561000000104	2172191000000114
8297491000006114	^ESCTED829749		education about anxiety	861611000000100	2228101000000119
14673781000006116	^ESCT1467378		generalized anxiety disorder 7 item score	445455005	4596987015
14673801000006117	^ESCT1467380		assessment using generalized anxiety disorder 7 item score	445598007	4596984010

Supplemental Table 2. Prescriptions codes used to define PNA.

ProdCodeId	dmdid	Term from EMIS	ProductName	DrugSubstanceName	BNFChapter
5813141000033117	16492311000001109	Alventa XL 150mg capsules (Consilient Health Ltd)	Alventa XL 150mg capsules	Venlafaxine hydrochloride	NA
5813341000033119	1649211100000110	Alventa XL 75mg capsules (Consilient Health Ltd)	Alventa XL 75mg capsules	Venlafaxine hydrochloride	NA

	7				
9300841000033114	24676911000001109	Amphero XL 150mg capsules (Viatris UK Healthcare Ltd)	Amphero XL 150mg capsules	Venlafaxine hydrochloride	NA
9300741000033116	24677311000001106	Amphero XL 75mg capsules (Viatris UK Healthcare Ltd)	Amphero XL 75mg capsules	Venlafaxine hydrochloride	NA
8265641000033113	21992211000001100	Bonilux XL 150mg capsules (Sandoz Ltd)	Bonilux XL 150mg capsules	Venlafaxine hydrochloride	NA
8265541000033112	21992411000001101	Bonilux XL 75mg capsules (Sandoz Ltd)	Bonilux XL 75mg capsules	Venlafaxine hydrochloride	NA
2749641000033115	3388511000001100	Cipralex 10mg tablets (Lundbeck Ltd)	Cipralex 10mg tablets	Escitalopram oxalate	NA
3928941000033119	10364011000001102	Cipralex 10mg/ml oral drops (Lundbeck Ltd)	Cipralex 10mg/ml oral drops	Escitalopram oxalate	NA
2951441000033110	4942511000001102	Cipralex 20mg tablets (Lundbeck Ltd)	Cipralex 20mg tablets	Escitalopram oxalate	NA
5128641000033118	15453311000001105	Cipralex 20mg/ml oral drops (Lundbeck Ltd)	Cipralex 20mg/ml oral drops	Escitalopram oxalate	NA
3030541000033115	7331811000001105	Cipralex 5mg tablets (Lundbeck Ltd)	Cipralex 5mg tablets	Escitalopram oxalate	NA
1583241000033111	62511000001109	Cipramil 10mg tablets (Lundbeck Ltd)	Cipramil 10mg tablets	Citalopram hydrobromide	NA
259141000033116	847211000001102	Cipramil 20mg tablets (Lundbeck Ltd)	Cipramil 20mg tablets	Citalopram hydrobromide	NA
1583041000033115	695611000001105	Cipramil 40mg tablets (Lundbeck Ltd)	Cipramil 40mg tablets	Citalopram hydrobromide	NA
1900741000033115	1211000001102	Cipramil 40mg/ml drops (Lundbeck Ltd)	Cipramil 40mg/ml drops	Citalopram hydrochloride	NA
1583341000033118	321989000	Citalopram 10mg tablets	Citalopram 10mg tablets	Citalopram hydrobromide	4030300
259241000033111	321987003	Citalopram 20mg tablets	Citalopram 20mg tablets	Citalopram hydrobromide	4030300
1583141000033116	321991008	Citalopram 40mg tablets	Citalopram 40mg tablets	Citalopram hydrobromide	4030300
1900641000033112	321994000	Citalopram 40mg/ml oral drops sugar free	Citalopram 40mg/ml oral drops sugar free	Citalopram hydrochloride	4030300
5896441000033118	17737011000001107	Depefex XL 150mg capsules (Chiesi Ltd)	Depefex XL 150mg capsules	Venlafaxine hydrochloride	NA
5896341000033112	17737211000001102	Depefex XL 75mg capsules (Chiesi Ltd)	Depefex XL 75mg capsules	Venlafaxine hydrochloride	NA
499041000033111	281211000001107	Efexor 37.5mg tablets (Wyeth Pharmaceuticals)	Efexor 37.5mg tablets	Venlafaxine hydrochloride	NA
499241000033115	628511000001107	Efexor 50mg tablets (Wyeth Pharmaceuticals)	Efexor 50mg tablets	Venlafaxine hydrochloride	NA
499141000033110	237411000001109	Efexor 75mg tablets (Wyeth Pharmaceuticals)	Efexor 75mg tablets	Venlafaxine hydrochloride	NA
498541000033113	413311000001104	Efexor XL 150mg capsules (Viatris UK Healthcare Ltd)	Efexor XL 150mg capsules	Venlafaxine hydrochloride	NA
10730141000033114	30991111000001106	Efexor XL 225mg capsules (Viatris UK Healthcare Ltd)	Efexor XL 225mg capsules	Venlafaxine hydrochloride	NA
498441000033112	651811000001100	Efexor XL 75mg capsules (Viatris UK Healthcare Ltd)	Efexor XL 75mg capsules	Venlafaxine hydrochloride	NA
2749541000033116	407915000	Escitalopram 10mg tablets	Escitalopram 10mg tablets	Escitalopram oxalate	4030300
3928841000033110	10364811000001108	Escitalopram 10mg/ml oral drops sugar free	Escitalopram 10mg/ml oral drops sugar free	Escitalopram oxalate	4030300
2951341000033116	408067001	Escitalopram 20mg tablets	Escitalopram 20mg tablets	Escitalopram oxalate	4030300
5128541000033119	15499311000001103	Escitalopram 20mg/ml oral drops sugar free	Escitalopram 20mg/ml oral drops sugar free	Escitalopram oxalate	4030300
3030341000033110	409150001	Escitalopram 5mg tablets	Escitalopram 5mg tablets	Escitalopram oxalate	4030300
561941000033115	595611000001101	Faverin 100mg tablets (Viatris UK Healthcare Ltd)	Faverin 100mg tablets	Fluvoxamine maleate	NA
563341000033115	542511000001103	Faverin 50mg tablets (Viatris UK Healthcare Ltd)	Faverin 50mg tablets	Fluvoxamine maleate	NA
1923141000033118	456311000001103	Felicium 20mg capsules (Opus Pharmaceuticals Ltd)	Felicium 20mg capsules	Fluoxetine hydrochloride	NA
11603841000033119	373959000	Fluoxetine 10mg capsules	Fluoxetine 10mg capsules	Fluoxetine hydrochloride	4030300
6496341000033116	373965000	Fluoxetine 10mg tablets	Fluoxetine 10mg tablets	Fluoxetine hydrochloride	4030300
13740541000033117	38962111000001102	Fluoxetine 10mg tablets (Imported)	Fluoxetine 10mg tablets	Fluoxetine hydrochloride	NA
577841000033114	321949006	Fluoxetine 20mg capsules	Fluoxetine 20mg capsules	Fluoxetine hydrochloride	4030300
9120641000033112	24407811000001108	Fluoxetine 20mg dispersible tablets sugar free	Fluoxetine 20mg dispersible tablets sugar free	Fluoxetine hydrochloride	4030300
2770641000033114	321951005	Fluoxetine 20mg/5ml oral solution	Fluoxetine 20mg/5ml oral solution	Fluoxetine hydrochloride	4030300
5749441000033115	11590211000001106	Fluoxetine 20mg/5ml oral solution sugar free	Fluoxetine 20mg/5ml oral solution sugar free	Fluoxetine hydrochloride	4030300
11707441000033119	32960811000001102	Fluoxetine 30mg capsules	Fluoxetine 30mg capsules	Fluoxetine hydrochloride	4030300
13740641000033116	38962311000001100	Fluoxetine 30mg capsules (Imported)	Fluoxetine 30mg capsules	Fluoxetine hydrochloride	NA
11707541000033118	376803002	Fluoxetine 40mg capsules	Fluoxetine 40mg capsules	Fluoxetine hydrochloride	4030300
579041000033111	321954002	Fluoxetine 60mg capsules	Fluoxetine 60mg capsules	Fluoxetine hydrochloride	4030300

592541000033118	NA	Fluoxetine Hydrochloride Liquid 20 mg/5 ml			NA
592841000033116	321947008	Fluvoxamine 100mg tablets	Fluvoxamine 100mg tablets	Fluvoxamine maleate	4030300
595541000033113	321945000	Fluvoxamine 50mg tablets	Fluvoxamine 50mg tablets	Fluvoxamine maleate	4030300
5234141000033115	15414711000001101	Foraven XL 150mg capsules (Forum Products Ltd)	Foraven XL 150mg capsules	Venlafaxine hydrochloride	NA
5234041000033119	15414911000001104	Foraven XL 75mg capsules (Forum Products Ltd)	Foraven XL 75mg capsules	Venlafaxine hydrochloride	NA
854141000033113	687611000001102	Lustral 100mg tablets (Viatris UK Healthcare Ltd)	Lustral 100mg tablets	Sertraline hydrochloride	NA
854241000033118	936411000001108	Lustral 50mg tablets (Viatris UK Healthcare Ltd)	Lustral 50mg tablets	Sertraline hydrochloride	NA
12901241000033119	36606711000001106	Majoven XL 150mg capsules (Bristol Laboratories Ltd)	Majoven XL 150mg capsules	Venlafaxine hydrochloride	NA
12901041000033110	36606311000001107	Majoven XL 37.5mg capsules (Bristol Laboratories Ltd)	Majoven XL 37.5mg capsules	Venlafaxine hydrochloride	NA
12901141000033114	36606511000001101	Majoven XL 75mg capsules (Bristol Laboratories Ltd)	Majoven XL 75mg capsules	Venlafaxine hydrochloride	NA
5889641000033110	17662411000001103	Mentaven XL 150mg capsules (Arrow Generics Ltd)	Mentaven XL 150mg capsules	Venlafaxine hydrochloride	NA
3029641000033112	375194009	Mirtazapine 15mg orodispersible tablets	Mirtazapine 15mg orodispersible tablets	Mirtazapine	4030400
3256741000033118	375193003	Mirtazapine 15mg tablets	Mirtazapine 15mg tablets	Mirtazapine	4030400
3058741000033113	9103511000001102	Mirtazapine 15mg/ml oral solution sugar free	Mirtazapine 15mg/ml oral solution sugar free	Mirtazapine	4030400
2974841000033114	375195005	Mirtazapine 30mg orodispersible tablets	Mirtazapine 30mg orodispersible tablets	Mirtazapine	4030400
919641000033115	321998002	Mirtazapine 30mg tablets	Mirtazapine 30mg tablets	Mirtazapine	4030400
3029741000033115	5627911000001100	Mirtazapine 45mg orodispersible tablets	Mirtazapine 45mg orodispersible tablets	Mirtazapine	4030400
3256841000033111	375196006	Mirtazapine 45mg tablets	Mirtazapine 45mg tablets	Mirtazapine	4030400
9120741000033115	24397511000001101	Olena 20mg dispersible tablets (Advanz Pharma)	Olena 20mg dispersible tablets	Fluoxetine hydrochloride	NA
2602041000033119	689411000001109	Oxactin 20mg capsules (Discovery Pharmaceuticals)	Oxactin 20mg capsules	Fluoxetine hydrochloride	NA
4079041000033115	39701911000001102	Paroxetine 10mg tablets	Paroxetine 10mg tablets	Paroxetine hydrochloride	4030300
1662441000033114	36565911000001109	Paroxetine 10mg/5ml oral suspension sugar free	Paroxetine 10mg/5ml oral suspension sugar free	Paroxetine hydrochloride	4030300
1042141000033117	321964006	Paroxetine 20mg tablets	Paroxetine 20mg tablets	Paroxetine hydrochloride	4030300
1042241000033112	321966008	Paroxetine 30mg tablets	Paroxetine 30mg tablets	Paroxetine hydrochloride	4030300
11755441000033119	422084009	Paroxetine 40mg tablets	Paroxetine 40mg tablets	Paroxetine hydrochloride	4030300
4957941000033115	15116511000001104	Politid XL 150mg capsules (Accord Healthcare Ltd)	Politid XL 150mg capsules	Venlafaxine hydrochloride	NA
4957841000033111	15116711000001109	Politid XL 75mg capsules (Accord Healthcare Ltd)	Politid XL 75mg capsules	Venlafaxine hydrochloride	NA
1112441000033114	573011000001101	Prozac 20mg capsules (Eli Lilly and Company Ltd)	Prozac 20mg capsules	Fluoxetine hydrochloride	NA
1129541000033110	695811000001109	Prozac 20mg/5ml liquid (Eli Lilly and Company Ltd)	Prozac 20mg/5ml liquid	Fluoxetine hydrochloride	NA
1111941000033111	142311000001103	Prozac 60mg capsules (Eli Lilly and Company Ltd)	Prozac 60mg capsules	Fluoxetine hydrochloride	NA
4498641000033115	11581211000001101	Prozep 20mg/5ml oral solution (Rosemont Pharmaceuticals Ltd)	Prozep 20mg/5ml oral solution	Fluoxetine hydrochloride	NA
2770541000033113	10065611000001109	Prozit 20mg/5ml oral solution (Pinewood Healthcare)	Prozit 20mg/5ml oral solution	Fluoxetine hydrochloride	NA
5329041000033111	16274611000001104	Ranfaxine XL 150mg capsules (Ranbaxy (UK) Ltd)	Ranfaxine XL 150mg capsules	Venlafaxine hydrochloride	NA
5328941000033119	16273411000001100	Ranfaxine XL 75mg capsules (Ranbaxy (UK) Ltd)	Ranfaxine XL 75mg capsules	Venlafaxine hydrochloride	NA
2710441000033119	683911000001104	Ranflutin 20mg capsules (Ranbaxy (UK) Ltd)	Ranflutin 20mg capsules	Fluoxetine hydrochloride	NA
4817341000033115	14934411000001108	Rodomel XL 150mg capsules (Teva UK Ltd)	Rodomel XL 150mg capsules	Venlafaxine hydrochloride	NA
4817441000033114	14934211000001109	Rodomel XL 75mg capsules (Teva UK Ltd)	Rodomel XL 75mg capsules	Venlafaxine hydrochloride	NA
4079141000033116	11270611000001109	Seroxat 10mg tablets (GlaxoSmithKline UK Ltd)	Seroxat 10mg tablets	Paroxetine hydrochloride	NA
1278341000033111	477511000001109	Seroxat 20mg tablets (GlaxoSmithKline UK Ltd)	Seroxat 20mg tablets	Paroxetine hydrochloride	NA
1686141000033119	16811000001109	Seroxat 20mg/10ml liquid (GlaxoSmithKline UK Ltd)	Seroxat 20mg/10ml liquid	Paroxetine hydrochloride	NA
1278441000033117	738111000001107	Seroxat 30mg tablets (GlaxoSmithKline UK Ltd)	Seroxat 30mg tablets	Paroxetine hydrochloride	NA
1278741000033112	39704011000001103	Sertraline 100mg tablets	Sertraline 100mg tablets	Sertraline hydrochloride	4030300
5975341000033119	8721711000001105	Sertraline 100mg/5ml oral suspension	Sertraline 100mg/5ml oral suspension	Sertraline hydrochloride	4030300
13743641000033113	39134011000001104	Sertraline 25mg tablets	Sertraline 25mg tablets	Sertraline hydrochloride	4030300

12900941000033117	13045911000001105	Sertraline 25mg/5ml oral suspension	Sertraline 25mg/5ml oral suspension	Sertraline hydrochloride	4030300
1278841000033119	39704111000001102	Sertraline 50mg tablets	Sertraline 50mg tablets	Sertraline hydrochloride	4030300
4148341000033116	8721911000001107	Sertraline 50mg/5ml oral suspension	Sertraline 50mg/5ml oral suspension	Sertraline hydrochloride	4030300
8298641000033115	21990711000001108	Sunveniz XL 150mg tablets (Sun Pharmaceutical Industries Europe B.V.)	Sunveniz XL 150mg tablets	Venlafaxine hydrochloride	NA
8298741000033112	21965311000001104	Sunveniz XL 75mg tablets (Sun Pharmaceutical Industries Europe B.V.)	Sunveniz XL 75mg tablets	Venlafaxine hydrochloride	NA
4897241000033112	14967611000001109	Tardcaps XL 150mg capsules (IXL Pharma Ltd)	Tardcaps XL 150mg capsules	Venlafaxine hydrochloride	NA
4897141000033117	14967411000001106	Tardcaps XL 75mg capsules (IXL Pharma Ltd)	Tardcaps XL 75mg capsules	Venlafaxine hydrochloride	NA
4817641000033111	14981711000001107	Tifaxin XL 150mg capsules (Genus Pharmaceuticals Ltd)	Tifaxin XL 150mg capsules	Venlafaxine hydrochloride	NA
4817741000033119	14981511000001102	Tifaxin XL 75mg capsules (Genus Pharmaceuticals Ltd)	Tifaxin XL 75mg capsules	Venlafaxine hydrochloride	NA
5574741000033114	16455211000001101	Tonpular XL 150mg capsules (Wockhardt UK Ltd)	Tonpular XL 150mg capsules	Venlafaxine hydrochloride	NA
5574641000033117	16455011000001106	Tonpular XL 75mg capsules (Wockhardt UK Ltd)	Tonpular XL 75mg capsules	Venlafaxine hydrochloride	NA
4937141000033116	15012111000001102	Vaxalin XL 150mg capsules (Ratiopharm UK Ltd)	Vaxalin XL 150mg capsules	Venlafaxine hydrochloride	NA
4937041000033115	15011711000001108	Vaxalin XL 75mg capsules (Ratiopharm UK Ltd)	Vaxalin XL 75mg capsules	Venlafaxine hydrochloride	NA
4936741000033119	15068911000001100	Venaxx XL 150mg capsules (Advanz Pharma)	Venaxx XL 150mg capsules	Venlafaxine hydrochloride	NA
4936641000033111	15068711000001102	Venaxx XL 75mg capsules (Advanz Pharma)	Venaxx XL 75mg capsules	Venlafaxine hydrochloride	NA
12194541000033118	34180811000001108	Vencarm XL 150mg capsules (Aspire Pharma Ltd)	Vencarm XL 150mg capsules	Venlafaxine hydrochloride	NA
12194641000033117	34180611000001109	Vencarm XL 225mg capsules (Aspire Pharma Ltd)	Vencarm XL 225mg capsules	Venlafaxine hydrochloride	NA
12194741000033114	34181211000001101	Vencarm XL 37.5mg capsules (Aspire Pharma Ltd)	Vencarm XL 37.5mg capsules	Venlafaxine hydrochloride	NA
12194841000033116	34181011000001106	Vencarm XL 75mg capsules (Aspire Pharma Ltd)	Vencarm XL 75mg capsules	Venlafaxine hydrochloride	NA
5812641000033119	16421511000001100	Venlablue XL 150mg capsules (Zentiva Pharma UK Ltd)	Venlablue XL 150mg capsules	Venlafaxine hydrochloride	NA
8885541000033117	23474511000001108	Venlablue XL 37.5mg capsules (Zentiva Pharma UK Ltd)	Venlablue XL 37.5mg capsules	Venlafaxine hydrochloride	NA
5812541000033115	16421311000001106	Venlablue XL 75mg capsules (Zentiva Pharma UK Ltd)	Venlablue XL 75mg capsules	Venlafaxine hydrochloride	NA
12605441000033117	23343211000001100	Venladex XL 150mg tablets (Dexcel-Pharma Ltd)	Venladex XL 150mg tablets	Venlafaxine hydrochloride	NA
12605541000033116	35638211000001107	Venladex XL 225mg tablets (Dexcel-Pharma Ltd)	Venladex XL 225mg tablets	Venlafaxine hydrochloride	NA
12605341000033111	23343411000001101	Venladex XL 75mg tablets (Dexcel-Pharma Ltd)	Venladex XL 75mg tablets	Venlafaxine hydrochloride	NA
1509241000033110	38750411000001106	Venlafaxine 150mg modified-release capsules	Venlafaxine 150mg modified-release capsules	Venlafaxine hydrochloride	4030400
4823641000033111	14984911000001107	Venlafaxine 150mg modified-release tablets	Venlafaxine 150mg modified-release tablets	Venlafaxine hydrochloride	4030400
10730041000033110	31016011000001103	Venlafaxine 225mg modified-release capsules	Venlafaxine 225mg modified-release capsules	Venlafaxine hydrochloride	4030400
5052741000033119	15439111000001107	Venlafaxine 225mg modified-release tablets	Venlafaxine 225mg modified-release tablets	Venlafaxine hydrochloride	4030400
13318341000033119	38003311000001102	Venlafaxine 300mg modified-release tablets	Venlafaxine 300mg modified-release tablets	Venlafaxine hydrochloride	4030400
8885441000033118	23560511000001101	Venlafaxine 37.5mg modified-release capsules	Venlafaxine 37.5mg modified-release capsules	Venlafaxine hydrochloride	4030400
6161741000033111	18765111000001101	Venlafaxine 37.5mg modified-release tablets	Venlafaxine 37.5mg modified-release tablets	Venlafaxine hydrochloride	4030400
1512241000033119	39707611000001104	Venlafaxine 37.5mg tablets	Venlafaxine 37.5mg tablets	Venlafaxine hydrochloride	4030400
6000441000033115	14017911000001109	Venlafaxine 37.5mg/5ml oral solution	Venlafaxine 37.5mg/5ml oral solution	Venlafaxine hydrochloride	4030400
13916241000033115	40111011000001106	Venlafaxine 37.5mg/5ml oral solution sugar free	Venlafaxine 37.5mg/5ml oral solution sugar free	Venlafaxine hydrochloride	4030400
4498541000033116	8722711000001103	Venlafaxine 37.5mg/5ml oral suspension	Venlafaxine 37.5mg/5ml oral suspension	Venlafaxine hydrochloride	4030400
1512441000033118	39707311000001109	Venlafaxine 50mg tablets	Venlafaxine 50mg tablets	Venlafaxine hydrochloride	4030400
1509341000033117	38750311000001104	Venlafaxine 75mg modified-release capsules	Venlafaxine 75mg modified-release capsules	Venlafaxine hydrochloride	4030400
4823541000033110	14985011000001107	Venlafaxine 75mg modified-release tablets	Venlafaxine 75mg modified-release tablets	Venlafaxine hydrochloride	4030400
1512341000033112	39707411000001102	Venlafaxine 75mg tablets	Venlafaxine 75mg tablets	Venlafaxine hydrochloride	4030400
5008041000033112	14018011000001106	Venlafaxine 75mg/5ml oral solution	Venlafaxine 75mg/5ml oral solution	Venlafaxine hydrochloride	4030400
13916341000033113	40111111000001107	Venlafaxine 75mg/5ml oral solution sugar free	Venlafaxine 75mg/5ml oral solution sugar free	Venlafaxine hydrochloride	4030400
8867941000033118	1401811100000110	Venlafaxine 75mg/5ml oral suspension	Venlafaxine 75mg/5ml oral	Venlafaxine hydrochloride	4030400

	7		suspension		
5052941000033116	15375411000001109	Venlalic XL 150mg tablets (Ethypharm UK Ltd)	Venlalic XL 150mg tablets	Venlafaxine hydrochloride	NA
5052841000033112	15376011000001109	Venlalic XL 225mg tablets (Ethypharm UK Ltd)	Venlalic XL 225mg tablets	Venlafaxine hydrochloride	NA
13318441000033113	37997111000001105	Venlalic XL 300mg tablets (Ethypharm UK Ltd)	Venlalic XL 300mg tablets	Venlafaxine hydrochloride	NA
6161841000033118	18748311000001101	Venlalic XL 37.5mg tablets (Ethypharm UK Ltd)	Venlalic XL 37.5mg tablets	Venlafaxine hydrochloride	NA
5053041000033114	15375711000001103	Venlalic XL 75mg tablets (Ethypharm UK Ltd)	Venlalic XL 75mg tablets	Venlafaxine hydrochloride	NA
6029641000033116	18148811000001100	Venlaneo XL 150mg capsules (Kent Pharma (UK) Ltd)	Venlaneo XL 150mg capsules	Venlafaxine hydrochloride	NA
6029741000033113	18148611000001104	Venlaneo XL 75mg capsules (Kent Pharma (UK) Ltd)	Venlaneo XL 75mg capsules	Venlafaxine hydrochloride	NA
12390341000033114	34740711000001101	Venlasov XL 150mg capsules (Sovereign Medical Ltd)	Venlasov XL 150mg capsules	Venlafaxine hydrochloride	NA
12389941000033119	34740911000001104	Venlasov XL 75mg capsules (Sovereign Medical Ltd)	Venlasov XL 75mg capsules	Venlafaxine hydrochloride	NA
4899641000033119	14975411000001107	Vensir XL 150mg capsules (Morningside Healthcare Ltd)	Vensir XL 150mg capsules	Venlafaxine hydrochloride	NA
11693641000033117	32833611000001107	Vensir XL 225mg capsules (Morningside Healthcare Ltd)	Vensir XL 225mg capsules	Venlafaxine hydrochloride	NA
4899541000033115	14975211000001108	Vensir XL 75mg capsules (Morningside Healthcare Ltd)	Vensir XL 75mg capsules	Venlafaxine hydrochloride	NA
12641541000033113	35797111000001102	Venzip XL 150mg capsules (Milpharm Ltd)	Venzip XL 150mg capsules	Venlafaxine hydrochloride	NA
12641641000033114	35797611000001105	Venzip XL 75mg capsules (Milpharm Ltd)	Venzip XL 75mg capsules	Venlafaxine hydrochloride	NA
4896741000033115	14974711000001100	Vexarin XL 150mg capsules (Viatris UK Healthcare Ltd)	Vexarin XL 150mg capsules	Venlafaxine hydrochloride	NA
4896841000033113	14974911000001103	Vexarin XL 75mg capsules (Viatris UK Healthcare Ltd)	Vexarin XL 75mg capsules	Venlafaxine hydrochloride	NA
4897041000033116	14978711000001108	ViePax 37.5mg tablets (Dexcel-Pharma Ltd)	ViePax 37.5mg tablets	Venlafaxine hydrochloride	NA
4896941000033117	14978911000001105	ViePax 75mg tablets (Dexcel-Pharma Ltd)	ViePax 75mg tablets	Venlafaxine hydrochloride	NA
4823841000033112	14981311000001108	ViePax XL 150mg tablets (Dexcel-Pharma Ltd)	ViePax XL 150mg tablets	Venlafaxine hydrochloride	NA
4823741000033119	14980711000001104	ViePax XL 75mg tablets (Dexcel-Pharma Ltd)	ViePax XL 75mg tablets	Venlafaxine hydrochloride	NA
4824641000033113	14954611000001107	Winfex XL 75mg capsules (Zentiva Pharma UK Ltd)	Winfex XL 75mg capsules	Venlafaxine hydrochloride	NA
1558041000033118	523611000001107	Zispin 30mg tablets (Organon Laboratories Ltd)	Zispin 30mg tablets	Mirtazapine	NA
3029841000033113	5608811000001102	Zispin SolTab 15mg orodispersible tablets (Organon Pharma (UK) Ltd)	Zispin SolTab 15mg orodispersible tablets	Mirtazapine	NA
3078541000033115	4859711000001106	Zispin SolTab 30mg orodispersible tablets (Organon Pharma (UK) Ltd)	Zispin SolTab 30mg orodispersible tablets	Mirtazapine	NA
3029941000033117	5595811000001101	Zispin SolTab 45mg orodispersible tablets (Organon Pharma (UK) Ltd)	Zispin SolTab 45mg orodispersible tablets	Mirtazapine	NA

Supplemental Figure-1. Adjusted incidence rates ratios for primary care clinical encounters in women with perinatal anxiety compared to matched unexposed cohorts over the study period

Adjusted for ethnicity, index of multiple deprivation, smoking, obesity status and Charlson comorbidity index

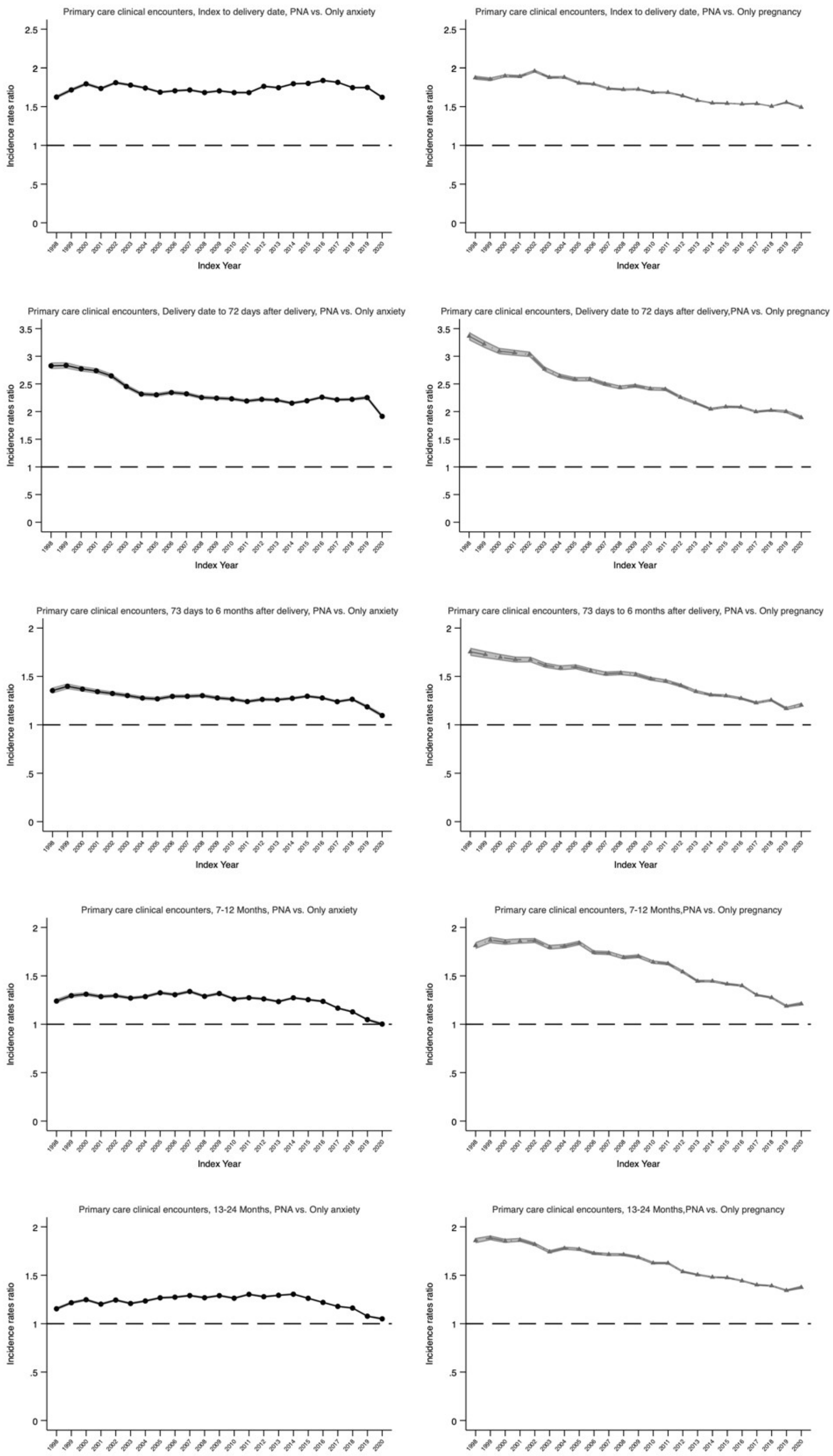
Primary care clinical encounters, Index to delivery date, PNA vs. Only anxiety
Primary care clinical encounters, Index to delivery date, PNA vs. Only pregnancy
Primary care clinical encounters, Delivery date to 72 days after delivery, PNA vs. Only anxiety
Primary care clinical encounters, Delivery date to 72 days after delivery,PNA vs. Only pregnancy
Primary care clinical encounters, 73 days to 6 months after delivery, PNA vs. Only anxiety
Primary care clinical encounters, 73 days to 6 months after delivery, PNA vs. Only pregnancy
Primary care clinical encounters, 7-12 Months, PNA vs. Only anxiety
Primary care clinical encounters, 7-12 Months,PNA vs. Only pregnancy
Primary care clinical encounters, 13-24 Months, PNA vs. Only anxiety
Primary care clinical encounters, 13-24 Months,PNA vs. Only pregnancy
Incidence rates ratio
Index Year

Supplemental Figure-2. Adjusted incidence rates ratio for referrals in women with perinatal anxiety compared to matched unexposed cohorts over the study period

Adjusted for ethnicity, index of multiple deprivation, smoking, obesity status and Charlson comorbidity index

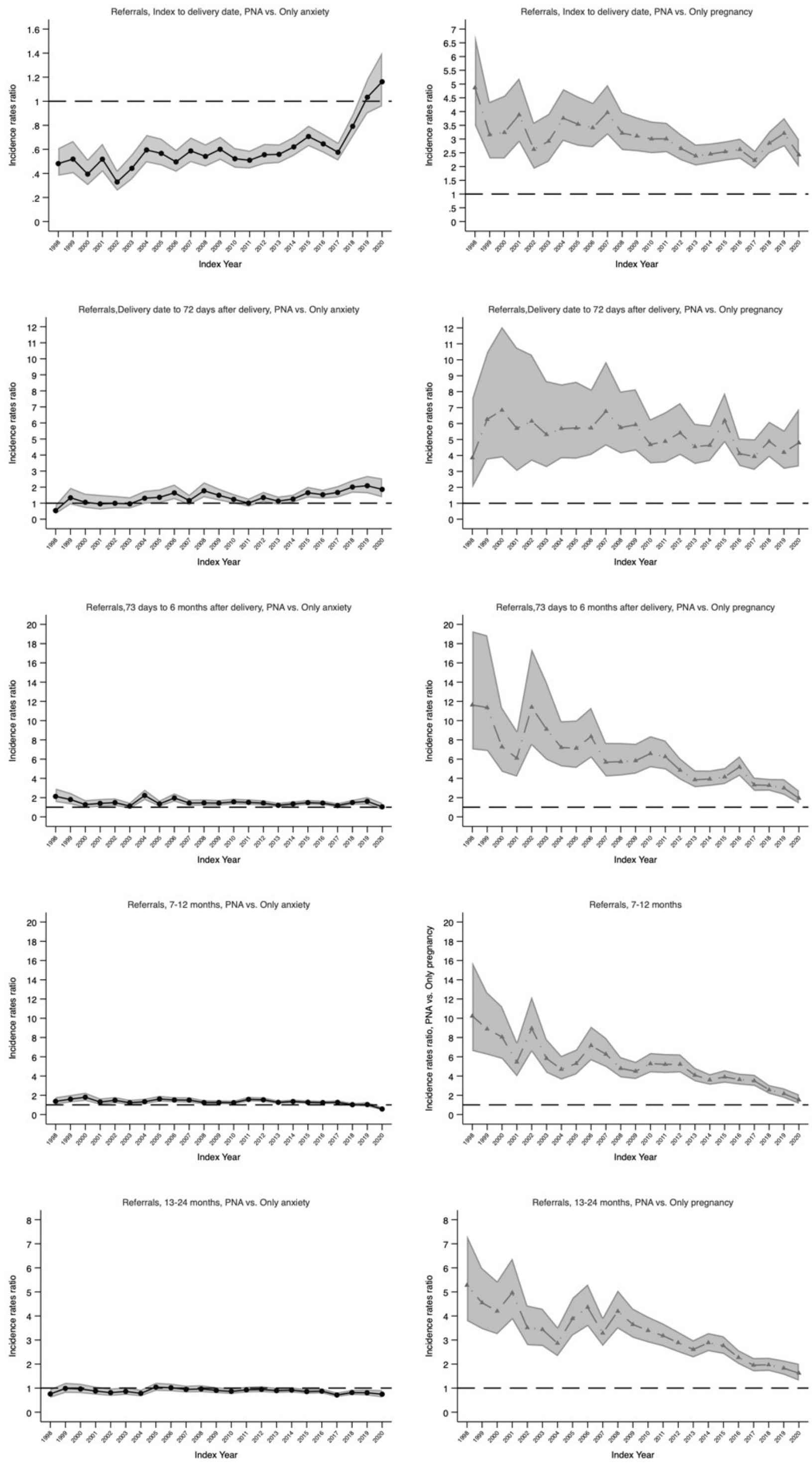

Supplemental Figure-3. Adjusted incidence rates ratio for prescriptions in women with perinatal anxiety compared to matched unexposed cohorts over the study period

Adjusted for ethnicity, index of multiple deprivation, smoking, obesity status and Charlson comorbidity index.

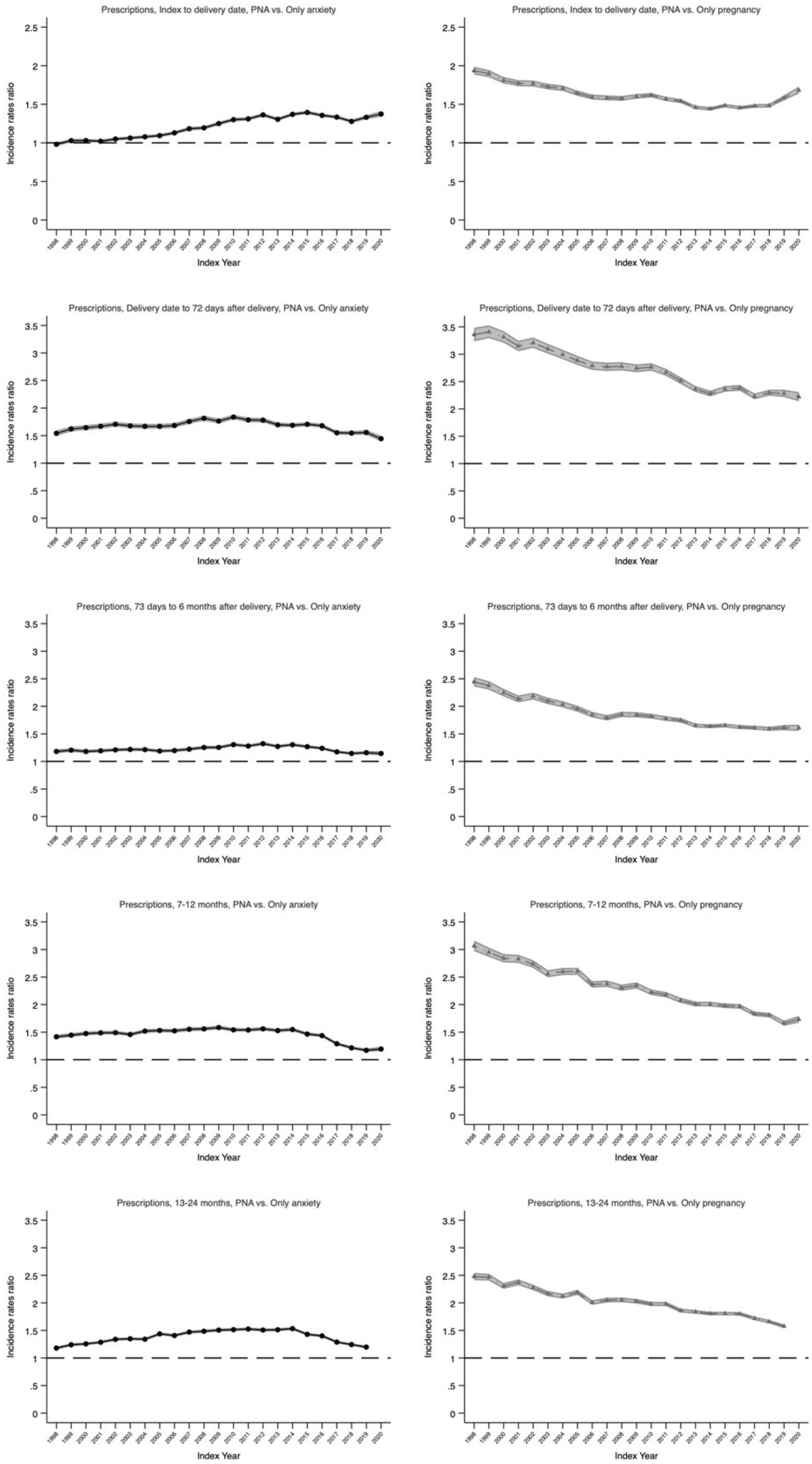

Supplemental Figure-4. Adjusted incidence rates ratio for hospitalisation between index date and 180 days after index date in women with perinatal anxiety compared to matched unexposed cohorts over the study period

Adjusted for ethnicity, index of multiple deprivation, smoking, obesity status and Charlson comorbidity index were adjusted.

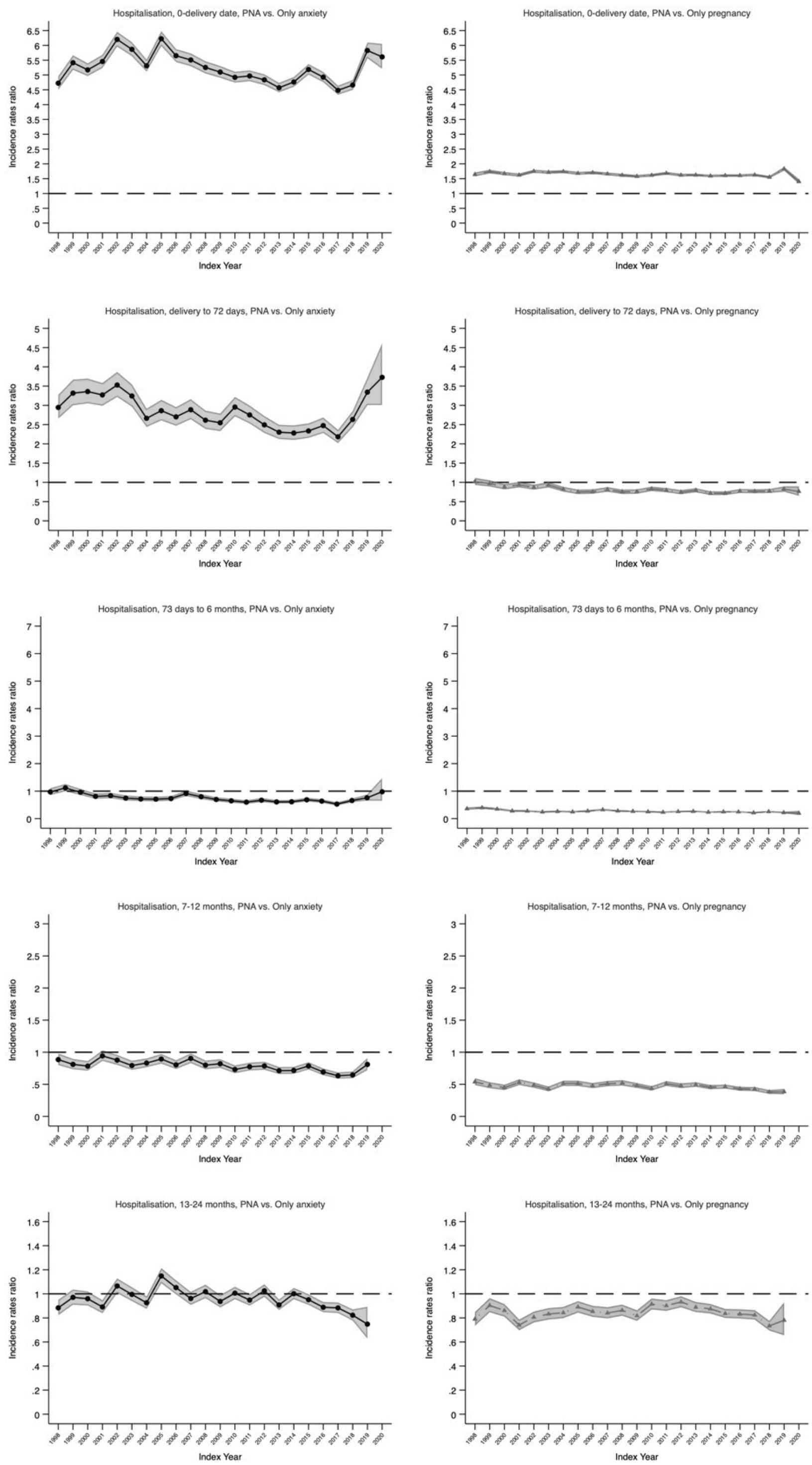

Supplemental Table-3. Unadjusted and adjusted incidence rates ratio (IRR) and 95% CI for healthcare utilisation

	Period-1: Index date-delivery date		Period-2: delivery date-72 days postpartum		Period-3: 73 days-6 months postpartum		Period-4: 7-12 months postpartum		Period-5: 13-24 months postpartum	
	Matched cohort 1* Anxiety without pregnancy	Matched cohort 2$ Pregnancy without anxiety	Matched cohort 1* Anxiety without pregnancy	Matched cohort 2$ Pregnancy without anxiety	Matched cohort 1* Anxiety without pregnancy	Matched cohort 2$ Pregnancy without anxiety	Matched cohort 1* Anxiety without pregnancy	Matched cohort 2$ Pregnancy without anxiety	Matched cohort 1* Anxiety without pregnancy	Matched cohort 2$ Pregnancy without anxiety
**Primary care clinical encounters**										
**Unadjusted**	1.845 (1.842 to 1.848)	1.719 (1.716 to 1.720)	2.394 (2.386 to 2.398)	2.394 (2.386 to 2.398)	1.335 (1.332 to 1.340)	1.457 (1.452 to 1.463)	1.305 (1.302 to 1.308)	1.589 (1.587 to 1.590)	1.306 (1.302 to 1.307)	1.632 (1.629 to 1.634)
**Adjusted**	1.742 (1.739 to 1.746)	1.653 (1.651 to 1.655)	2.288 (2.283 to 2.293)	2.319 (2.314 to 2.324)	1.270 (1.266 to 1.273)	1.405 (1.401 to 1.408)	1.237 (1.234 to 1.240)	1.533 (1.531 to 1.536)	1.229 (1.227 to 1.231)	1.569 (1.567 to 1.571)
**Referrals**										
**Unadjusted**	0.644 (0.624 to 0.666)	2.954 (2.841 to 3.077)	1.491 (1.417 to 1.566)	5.076 (4.762 to 5.405)	1.528 (1.466 to 1.595)	5.022 (4.762 to 5.291)	1.386 (1.339 to 1.435)	4.479 (4.291 to 4.673)	0.933 (0.906 to 0.962)	2.932 (2.833 to 3.039)
**Adjusted**	0.600 (0.581 to 0.621)	2.841 (2.725 to 2.949)	1.437 (1.364 to 1.513)	4.902 (4.608 to 5.236)	1.440 (1.378 to 1.503)	4.831 (4.587 to 5.102)	1.310 (1.264 to 1.358)	4.310 (4.115 to 4.483)	0.874 (0.849 to 0.902)	2.817 (2.719 to 2.918)
**Prescriptions**										
**Unadjusted**	1.287 (1.282 to 1.290)	1.672 (1.666 to 1.678)	1.724 (1.715 to 1.732)	2.761 (2.747 to 2.778)	1.274 (1.268 to 1.280)	1.912 (1.901 to 1.923)	1.508 (1.502 to 1.515)	2.347 (2.336 to 2.358)	1.445 (1.438 to 1.452)	2.071 (2.058 to 2.080)
**Adjusted**	1.203 (1.199 to 1.209)	1.582 (1.572 to 1.585)	1.650 (1.641 to 1.658)	2.611 (2.597 to 2.625)	1.211 (1.205 to 1.217)	1.802 (1.796 to 1.815)	1.458 (1.451 to 1.464)	2.232 (2.220 to 2.242)	1.379 (1.373 to 1.384)	1.969 (1.961 to 1.976)
**Hospitalisations**										
**Unadjusted**	5.844 (5.814 to 5.882)	1.752 (1.741 to 1.763)	3.075 (3.021 to 3.135)	0.853 (0.840 to 0.866)	0.813 (0.794 to 0.833)	0.284 (0.278 to 0.290)	0.890 (0.874 to 0.906)	0.503 (0.495 to 0.512)	1.101 (1.088 to 1.113)	0.913 (0.904 to 0.923)
**Adjusted**	5.155 (5.128 to 5.182)	1.637 (1.626 to 1.648)	2.732 (2.681 to 2.780)	0.805 (0.792 to 0.817)	0.722 (0.705 to 0.740)	0.265 (0.260 to 0.271)	0.782 (0.768 to 0.796)	0.469 (0.461 to 0.477)	0.960 (0.950 to 0.972)	0.848 (0.839 to 0.859)

Adjusted for ethnicity, index of multiple deprivation (IMD), smoking status, obesity, and Charlson comorbidity index.
* Indicates the matched cohort 1 (anxiety without pregnancy) was used as the reference group.
$ indicates the matched cohort 2 (pregnancy without anxiety) was used as the reference group.
CI indicates confidence interval.

Supplemental Table-4. Unadjusted and adjusted absolute risk difference (ARD) per 1,000 person-years (95% CI) for healthcare utilisation

	Period-1: Index date-delivery date		Period-2: delivery date-72 days postpartum		Period-3: 73 days -6 months postpartum		Period 4: 7-12 months postpartum		Period-5: 13-24 months postpartum	
	Matched cohort 1* Anxiety without pregnancy	Matched cohort 2$ Pregnancy without anxiety	Matched cohort 1* Anxiety without pregnancy	Matched cohort 2$ Pregnancy without anxiety	Matched cohort 1* Anxiety without pregnancy	Matched cohort 2$ Pregnancy without anxiety	Matched cohort 1* Anxiety without pregnancy	Matched cohort 2$ Pregnancy without anxiety	Matched cohort 1* Anxiety without pregnancy	Matched cohort 2$ Pregnancy without anxiety
**Primary care clinical encounters**										
**Unadjusted**	8409.1 (8381.8 to 8436.3)	7676.0 (7648.7 to 7703.4)	3924.5 (3908.2 to 3940.8)	3924.5 (3908.2 to 3940.8)	1376.4 (1361.2 to 1391.6)	1718.9 (1703.8 to 1734.0)	1963.2 (1944.3 to 1982.0)	3113.2 (3094.6 to 3131.8)	3580.8 (3555.3 to 3606.2)	5916.3 (5891.2 to 5941.3)
**Adjusted**	7623.6 (7596.8 to 7650.4)	6849.1 (6822.4 to 6875.7)	3707.5 (3691.3 to 3723.6)	3690.9 (3674.9 to 3706.8)	1180.1 (1165.1 to 1195.2)	1497.2 (1482.4 to 1512.0)	1625.4 (1606.8 to 1644.1)	2756.7 (2738.6 to 2774.8)	2847.0 (2822.0 to 2872.0)	5222.9 (5198.6 to 5247.2)
**Referrals**										
**Unadjusted**	-21.7 (-23.1 to -20.3)	26.0 (24.8 to 27.3)	6.3 (5.4 to 7.2)	15.4 (14.6 to 16.3)	9.5 (8.5 to 10.6)	22.1 (21.1 to 23.1)	11.3 (10.0 to 12.6)	31.6 (30.3 to 32.8)	-3.6 (-5.1 to -2.1)	33.4 (32.0 to 34.7)
**Adjusted**	-22.9 (-24.3 to -21.5)	24.9 (23.6 to 26.1)	5.7 (4.8 to 6.6)	14.8 (14.0 to 15.7)	8.6 (7.5 to 9.7)	21.2 (20.2 to 22.2)	10.2 (8.9 to 11.5)	30.3 (29.1 to 31.5)	-5.2 (-6.7 to -3.6)	31.7 (30.3 to 33.0)
**Prescriptions**										
**Unadjusted**	745.9 (734.0 to 757.8)	1346.6 (1334.9 to 1358.3)	861.5 (852.4 to 870.6)	1308.1 (1299.2 to 1317.0)	404.9 (396.0 to 413.9)	897.8 (889.1 to 906.5)	775.5 (765.7 to 785.3)	1322.3 (1312.8 to 1331.8)	923.5 (912.3 to 934.7)	1547.9 (1537.0 to 1558.9)
**Adjusted**	536.7 (525.1 to 548.4)	1164.4 (1153.1 to 1175.6)	772.3 (763.4 to 781.3)	1209.8 (1201.2 to 1218.5)	312.3 (303.6 to 321.1)	800.5 (792.1 to 808.9)	668.5 (658.9 to 678.1)	1208.8 (1199.5 to 1218.0)	752.1 (741.2 to 763.1)	1395.5 (1384.9 to 1406.0)
**Hospitalisations**										
**Unadjusted**	1145.0 (1137.9 to 1152.2)	593.2 (585.7 to 600.7)	125.7 (123.0 to 128.4)	-32.1 (-35.0 to -29.2)	-17.4 (-19.3 to -15.6)	-191.3 (-193.6 to -189.1)	-17.1 (-19.7 to -14.6)	-136.7 (-139.4 to -134.0)	31.2 (27.3 to 35.0)	-32.3 (-36.3 to -28.4)
**Adjusted**	1023.8 (1017.1 to 1030.5)	493.7 (486.7 to 500.7)	109.1 (106.6 to 111.6)	-41.9 (-44.6 to -39.1)	-27.4 (-29.2 to -25.6)	-192.6 (-194.8 to -190.4)	-36.3 (-38.6 to -33.9)	-143.6 (-146.2 to -141.1)	-15.4 (-19.1 to -11.8)	-56.6 (-60.2 to -53.0)

Adjusted for ethnicity, index of multiple deprivation (IMD), smoking status, obesity, and Charlson comorbidity index.
* Indicates the matched cohort 1 (only anxiety) was used as the reference group.
$ indicates the matched cohort 2 (only pregnancy) was used as the reference group.
CI indicates confidence interval.

Supplemental Table-5. Adjusted incidence rates ratio (IRR) and absolute risk difference (ARD) for healthcare utilisation in the complete cases

	Period-1: Index date-delivery date		Period-2: delivery date-72 days postpartum		Period-3: 73 days-6 months postpartum		Period-4: 7-12 months postpartum		Period-5: 13-24 months postpartum	
	Matched cohort 1* Anxiety without pregnancy	Matched cohort 2$ Pregnancy without anxiety	Matched cohort 1* Anxiety without pregnancy	Matched cohort 2$ Pregnancy without anxiety	Matched cohort 1* Anxiety without pregnancy	Matched cohort 2$ Pregnancy without anxiety	Matched cohort 1* Anxiety without pregnancy	Matched cohort 2$ Pregnancy without anxiety	Matched cohort 1* Anxiety without pregnancy	Matched cohort 2$ Pregnancy without anxiety
**Primary care clinical encounters**										
**Adjusted IRR**	1.74 (1.73 to 1.75)	1.65 (1.64 to 1.66)	2.29 (2.28 to 2.30)	2.32 (2.31 to 2.33)	1.27 (1.26 to 1.28)	1.41 (1.40 to 1.42)	1.24 (1.23 to 1.25)	1.53 (1.52 to 1.54)	1.22 (1.21 to 1.23)	1.57 (1.56 to 1.58)
**Adjusted ARD**	7623.5 (7596.7 to 7650.2)	6848.0 (6821.3 to 6876.3)	3706.3 (3691.3 to 3723.6)	3686.6 (3670.3 to 3703.5)	1178.6 (1163.5 to 1193.6)	1496.3 (1480.3 to 1510.6)	1623.6 (1603.5 to 1639.6)	2753.6 (2736.3 to 2770.3)	2842.6 (2816.6 to 2869.7)	5220.8 (5196.3 to 5243.5)
**Referrals**										
**Adjusted IRR**	0.60 (0.58 to 0.62)	2.84 (2.73 to 2.95)	1.44 (1.36 to 1.52)	4.90 (4.61 to 5.23)	1.43 (1.37 to 1.50)	4.82 (4.59 to 5.10)	1.31 (1.26 to 1.36)	4.31 (4.12 to 4.48)	0.87 (0.85 to 0.90)	2.82 (2.72 to 2.92)
**Adjusted ARD**	-21.6 (-23.4 to -22.6)	25.2 (23.3 to 26.3)	5.6 (4.7 to 6.5)	14.6 (13.6 to 15.6)	8.3 (7.2 to 9.6)	21.0 (19.6 to 22.3)	10.1 (8.8 to 11.6)	30.1 (28.9 to 31.6)	-5.0 (-6.8 to -3.3)	31.3 (30.0 to 32.9)
**Prescriptions**										
**Adjusted IRR**	1.20 (1.18 to 1.22)	1.58 (1.57 to 1.59)	1.65 (1.64 to 1.66)	2.61 (2.59 to 2.63)	1.21 (1.20 to 1.22)	1.81 (1.80 to 1.82)	1.46 (1.45 to 1.47)	2.23 (2.22 to 2.24)	1.38 (1.37 to 1.39)	1.97 (1.96 to 1.98)
**Adjusted ARD**	535.6 (523.9 to 546.9)	1163.9 (1152.9 to 1173.9)	770.6 (762.6 to 780.3)	1206.8 (1200.6 to 1219.8)	311.9 (302.9 to 322.6)	800.6 (791.9 to 809.8)	669.8 (659.8 to 680.6)	1209.6 (1200.6 to 1220.8)	753.6 (740.6 to 765.6)	1396.8 (1383.6 to 1403.5)
**Hospitalisations**										
**Adjusted IRR**	5.16 (5.13 to 5.18)	1.64 (1.63 to 1.65)	2.73 (2.68 to 2.78)	0.81 (0.79 to 0.82)	0.72 (0.71 to 0.74)	0.27 (0.26 to 0.28)	0.78 (0.76 to 0.80)	0.47 (0.46 to 0.48)	0.96 (0.95 to 0.97)	0.85 (0.84 to 0.86)
**Adjusted ARD**	1022.6 (1016.6 to 1032.7)	495.2 (486.3 to 501.7)	110.2 (105.8 to 112.3)	-41.6 (-43.9 to -40.2)	-28.3 (-28.6 to -25.8)	-193.6 (-195.6 to -190.1)	-36.2 (-38.3 to -33.5)	-143.9 (-146.8 to -140.2)	-15.0 (-18.7 to -11.6)	-56.5 (-60.6 to -52.1)

Adjusted for ethnicity, index of multiple deprivation (IMD), smoking status, obesity, and Charlson comorbidity index.
* Indicates the matched cohort 1 (anxiety without pregnancy) was used as the reference group.
$ indicates the matched cohort 2 (pregnancy without anxiety) was used as the reference group.

Supplemental Table-6. Sensitivity analyses for the primary outcome: period-specific primary care clinical encounters rate ratios comparing cohorts, using clustered Poisson and population-averaged GEE Poisson models.

	PNA vs matched cohort 1 (Anxiety without pregnancy) IRR (clustered Poisson), 95% CI	PNA vs matched cohort 1 (Anxiety without pregnancy) IRR (GEE), 95% CI	PNA vs matched cohort 2 (Pregnancy without anxiety) IRR (clustered Poisson), 95% CI	PNA vs matched cohort 2 (Pregnancy without anxiety) IRR (GEE), 95% CI
**Period-1: Index date-delivery date**	1.766 (1.756, 1.775)	1.685 (1.676, 1.694)	1.641 (1.633, 1.648)	1.532 (1.524, 1.539)
**Period-2: delivery date-72 days postpartum**	2.290 (2.276, 2.304)	2.150 (2.136, 2.164)	2.285 (2.273, 2.297)	2.027 (2.015, 2.039)
**Period-3: 73 days-6 months postpartum**	1.277 (1.268, 1.286)	1.175 (1.167, 1.184)	1.390 (1.381, 1.399)	1.223 (1.215, 1.232)
**Period-4: 7-12 months postpartum**	1.248 (1.240, 1.257)	1.164 (1.156, 1.171)	1.516 (1.507, 1.526)	1.368 (1.359, 1.376)
**Period-5: 13-24 months postpartum**	1.249 (1.241, 1.257)	1.182 (1.175, 1.189)	1.558 (1.548, 1.567)	1.444 (1.435, 1.453)

IRRs compare primary care clinical encounter rates in the perinatal anxiety (PNA) cohort versus each matched comparator cohort within the same time period.
Clustered Poisson models used robust (sandwich) standard errors clustered at the woman level to account for within-person correlation across repeated period contributions (and multiple pregnancies where applicable).
Population-averaged Poisson GEE models used a log link, an exchangeable working correlation structure, and robust variance estimates.
All models included a log(person-time) offset (person-time in years) and were adjusted for ethnicity, deprivation decile, alcohol use, smoking status, obesity, and Charlson Comorbidity Index, consistent with the main analysis.
Period-specific IRRs were obtained from cohort-by-period interaction terms.
IRR>1 indicates higher encounter rates in the PNA cohort than in the matched comparator cohort for that period.
