# Supplementary material for: Impact of perinatal anxiety on healthcare utilisation: a matched cohort study using electronic health records in the UK
Source: BMJ Open. 2026 Sep 25;16(9):e100543. doi: 10.1136/bmjopen-2025-100543 (PMC13629954; doi:10.1136/bmjopen-2025-100543)
Supplement: Supplementary file 2 [file bmjopen-2025-100543.reviewer_comments.pdf]

PEER REVIEW HISTORY

BMJ Open publishes all reviews undertaken for accepted manuscripts. Reviewers are asked to complete a checklist review form and are provided with free text boxes to elaborate on their assessment. These free text comments are reproduced below.

ARTICLE DETAILS

Title (Provisional)

Impact of perinatal anxiety on healthcare utilisation: a matched cohort study using electronic health record in the United Kingdom

Authors

Yu, Dahai; Bailey, James; Chew-Graham, Carolyn A.; Smith, Holly Christina; Archer, Charlotte; Evans, Jonathan; Fisher, Tamsin; Kessler, David; Kingstone, Tom; Petersen, Irene; Procter, Janine; Shivji, Noureen; Silverwood, Victoria; Spruce, Amy; Turner, Katrina M; Wu, Pensee; Blagojevic-Bucknall, Milica; Jordan, Kelvin P

VERSION 1 - REVIEW

**Reviewer**	**1**
**Name**	**Saha, Sukanta**
**Affiliation**	**The Park Centre for Mental Health, Queendland Centre for Mental Health Research**
**Date**	**11-Nov-2025**
**COI**	**None**

Title:

Impact of perinatal anxiety on healthcare utilisation: a matched cohort study using electronic health record in the United Kingdom

General comment

The study is a retrospective cohort design using electronic health records to examine differences in healthcare utilisation patterns among women with perinatal anxiety (PNA) compared to women with anxiety but not pregnant and women with pregnancy but no anxiety. The main result highlights that women with PNA exhibit higher healthcare utilisation in relation to comparison groups.

The design was appropriate to examine the research questions. While it is not unknown that healthcare utilisation would be higher in prenatal, the study extended findings to the postpartum period. The results are important for health care system and support services. However, I have some specific suggestions to improve the manuscript further.

Specific comment

Abstract and Introduction

1. Page 2. Line 28: The tern like 'primary care encounter' may be very much familiar with primary care providers, it is better to defined briefly, (eg., number of doctor's visit?)
2. Page 4, Lines 38-52: Literature review looks very weak. There are plenty of studies to show that healthcare utilisation has always been higher in perinatal period. Refs 8 and 10 (Kingston et al 2012; Kiviruusu et al 2020) only showed psychological distress in this period, readers may be interested to see a comprehensive review about it. The impact of COVID-19 pandemic on maternal health is irrelevant in this context (Ref 9).

Methods

1. Methods look good. However, covariates were not defined well enough. For example, 'deprivation' using IMD is not well-defined. Similarly, readers may enquire about Charlson comorbidity index. Does comorbidity mean psychological problems other than anxiety? While the study has good strengths, psychological therapy use is an important consideration although data were not available.
2. Some of the abbreviated terms needs to be spelt out (eg., SSRI, CLASP, SNOMED etc)

Results

1. The results section may need to divide into paragraphs to increase readability either by Period 1, Period 2, etc or some other ways
2. If I understand correctly, one of your objectives is to show variation of healthcare utilisation patterns between primary and secondary healthcare services (page 4, lines 43-45) but that are not highlighted.
3. Authors conducted sensitivity analyses which is very well justified. However, statement like, "Sensitivity analysis using complete cases only gave similar findings as the main analysis" without any data is very weak.

Discussion and References

1. As mentioned there could be many more studies to show consistent results with the present study.
2. References cited in page 12, Lines 42, 49, 59 are all wrong. Sometimes PMIDs are mentioned (page 12, Lines 24, 54). The manuscript needs thorough checking of the reference system.

Tables and Figures

There are minor errors with a suggestion that all of them should be self-explanatory. Footnotes could be used to explain abbreviated texts, and better resolution for figures are expected.

Reviewer 2

Name Rampilo, Mashudu

Affiliation University of Johannesburg - Auckland Park Bunting Road Campus, Environmental Health

Date 25-Nov-2025

COI None

The paper examined differences in healthcare utilization among women with perinatal anxiety compared to two matched cohorts using electronic health records from CPRD.

I have included my specific comments on the document

**** The reviewer provided a marked copy with additional comments. Please contact the publisher for full details. ****

Reviewer 3

Name Zhuang , Yaxu

Affiliation University of Colorado Denver Anschutz Medical Campus, Department of Medicine

Date 09-Dec-2025

COI None

The manuscript is generally well written and clearly presented. However, there is a major concern regarding the statistical methodology used for the analysis of longitudinal outcomes. The authors estimated outcome rates within each follow-up period using Poisson regression models and evaluated associations between PNA and outcomes using conditional Poisson regression, both unadjusted and adjusted for ethnicity, geographic region, deprivation, comorbidity, obesity, and smoking status.

Given that each individual contributes repeated measurements across multiple follow-up periods, the data are inherently longitudinal and correlated. As such, the use of a generalized linear mixed model (GLMM), with appropriate random effects to account for within-subject correlation, would be more statistically appropriate than fitting separate Poisson models for each period.

Moreover, while the authors state that the longitudinal nature of the data allows examination of period effects and how the association between PNA and healthcare outcomes evolves over time, this claim is not adequately supported by the current modeling strategy. A GLMM framework with interaction terms between time period and PNA would more rigorously demonstrate these longitudinal and period-specific effects and provide a stronger basis for the authors' conclusions.

VERSION 1 - AUTHOR RESPONSE

Reviewer: 1
Dr. Sukanta Saha, The Park Centre for Mental Health
Comments to the Author:
Reviewers comment
Manuscript ID: bmjopen-2025-100543
Title:

Impact of perinatal anxiety on healthcare utilisation: a matched cohort study using electronic health record in the United Kingdom

General comment

The study is a retrospective cohort design using electronic health records to examine differences in healthcare utilisation patterns among women with perinatal anxiety (PNA) compared to women with anxiety but not pregnant and women with pregnancy but no anxiety. The main result highlights that women with PNA exhibit higher healthcare utilisation in relation to comparison groups.

The design was appropriate to examine the research questions. While it is not unknown that healthcare utilisation would be higher in prenatal, the study extended findings to the postpartum period. The results are important for health care system and support services. However, I have some specific suggestions to improve the manuscript further.

Specific comment

Abstract and Introduction

1. Page 2. Line 28: The tern like 'primary care encounter' may be very much familiar with primary care providers, it is better to defined briefly, (eg., number of doctor's visit?)

Response: Thank you. We agree this term should be clearer for an international readership. We have added a brief definition in the Abstract and Methods. Specifically, we now define primary care clinical encounters as recorded primary care consultations/contacts captured in CPRD primary care records (eg, GP or other primary care clinician contacts), rather than limiting this to "doctor visits" only. We revised the Abstract (Page 2, Line 28) as "Rates of primary care clinical encounters (recorded primary care consultations/contacts, including GP and other primary care clinician contacts), prescriptions, referrals, and hospitalisations." and added the corresponding clarification in the Outcomes section (Page 7, Lines 26–33) as: "The primary outcomes for this study were healthcare utilisation measured by the number of primary care clinical encounters (recorded primary care consultations/contacts in primary care records, including GP and other primary care clinician contacts), …"

2. Page 4, Lines 38-52: Literature review looks very weak. There are plenty of studies to show that healthcare utilisation has always been higher in perinatal period. Refs 8 and 10 (Kingston et al 2012; Kiviruusu et al 2020) only showed psychological distress in this period, readers may be interested to see a comprehensive review about it. The impact of COVID-19 pandemic on maternal health is irrelevant in this context (Ref 9).

Response: Thank you. We agree that the Introduction should better reflect evidence on healthcare utilisation across pregnancy and postpartum. We have therefore strengthened this paragraph by replacing citations that primarily address psychological distress or COVID-19 context with studies that directly describe primary care consultation patterns after childbirth, and by clarifying the specific evidence gaps motivating our study (limited focus on PNA, lack of matched comparisons, and limited evidence across care settings). We also removed the COVID-19-focused content and citations from this section.

We revised the paragraph in the Introduction (Page 4, Lines 38–52) to:

"While previous research acknowledges increased healthcare demands among women with PNA, critical gaps remain in our understanding of the long-term trajectories of healthcare utilisation patterns beyond the immediate postnatal period[7]. This includes a lack of detailed knowledge about how these patterns vary across primary and secondary healthcare services [8,9]. Although UK primary care studies have described marked changes in consultation patterns across pregnancy and the early postnatal period, with service use stabilising after the first months postpartum [7,8], these studies did not focus on women with PNA or provide matched comparisons. Moreover, there is limited understanding of how women with PNA engage with healthcare services across different socio-demographic and ethnic groups, suggesting a need for more focused studies in this area [9,10]."

The updated 7-10th references were listed as below:

7. Smith HC, Saxena S, Petersen I. Postnatal checks and primary care consultations in the year following childbirth: an observational cohort study of 309 573 women in the UK, 2006–2016. BMJ Open 2020;10:e036835.

8. Smith HC, Schartau P, Saxena S, Petersen I. The first 100 days after childbirth: cross-sectional study of maternal clinical events and health needs from primary care. Br J Gen Pract 2024;74:e580–e586.

9. Howard LM, Khalifeh H. Perinatal mental health: a review of progress and challenges. World Psychiatry2020;19:313–327.

10. Pisavadia K, Spencer LH, Tuersley L, Coates R, Ayers S, Edwards RT. Health economic evaluations of preventative care for perinatal anxiety and associated disorders: a rapid review. BMJ Open 2024;14:e068941.

Methods

1. Methods look good. However, covariates were not defined well enough. For example, 'deprivation' using IMD is not well-defined. Similarly, readers may enquire about Charlson comorbidity index. Does comorbidity mean psychological problems other than anxiety? While the study has good strengths, psychological therapy use is an important consideration although data were not available.

Response: Thank you. We agree the covariate definitions needed to be clearer for an international readership. We have therefore clarified (i) what the Index of Multiple Deprivation (IMD) represents and how we grouped it, and (ii) that the Charlson Comorbidity Index in this study reflects pre-existing physical comorbidity burden (and does not represent "psychological comorbidity"). We also explicitly acknowledge that psychological therapy use is not comprehensively captured in CPRD/HES and therefore could not be adjusted for, and we have added this as a limitation.

We revised the covariates description in the Methods (Page 9, Lines 17–40) to:

"Neighbourhood deprivation level was categorised based on IMD (an area-level measure of relative socioeconomic deprivation in England). This is a composite measure covering seven domains (health deprivation and disability; barriers to housing and services; employment; income; education, skills and training; crime; living environment). IMD was categorised into

deciles (IMD 1=least deprived to IMD 10=most deprived), with an additional category for individuals without IMD linkage.
Comorbidity status was dichotomised as no comorbidity (0) versus comorbidity (≥1) based on Charlson comorbidity index, which summarises pre-existing chronic physical conditions recorded prior to the index date and does not represent psychological comorbidity."

We also revised the Limitations paragraph (Page 15, Lines 27–35) to include:

"In addition, use of psychological therapies (eg, talking therapies/IAPT) is not comprehensively recorded in primary care; therefore, we could not adjust for receipt of non-pharmacological treatment."

2. Some of the abbreviated terms needs to be spelt out (eg., SSRI, CLASP, SNOMED etc)

Response: Thank you. We agree and have expanded key abbreviations at first mention in the manuscript (and retained abbreviations thereafter for readability). Specifically, we have defined selective serotonin reuptake inhibitors (SSRIs), Clinical Practice Research Datalink (CPRD), Systematised Nomenclature of Medicine—Clinical Terms (SNOMED CT), Hospital Episode Statistics (HES), Index of Multiple Deprivation (IMD), and Clinical Practice Research Datalink (CPRD) Pregnancy Register at first mention. We have also added a short definition for the CLASP program name at first mention.

We revised the relevant text in the Methods/Study data source and exposure definition sections to:

"We used the Clinical Practice Research Datalink (CPRD) Aurum … linked to Hospital Episode Statistics (HES) and the Index of Multiple Deprivation (IMD) …"

"Perinatal anxiety was identified using recorded anxiety diagnoses coded using Systematised Nomenclature of Medicine—Clinical Terms (SNOMED CT) and/or prescriptions for anxiolytic drugs or selective serotonin reuptake inhibitors (SSRIs) …"

"This study was conducted within the CLAssification and outcomeS of Perinatal anxiety (CLASP) program …"

Results

1. The results section may need to divide into paragraphs to increase readability either by Period 1, Period 2, etc or some other ways

Response: Thank you. We agree and have reorganised the Results to improve readability by structuring the main findings by follow-up period (Period-1 to Period-5), with short standalone paragraphs for each time window.

2. If I understand correctly, one of your objectives is to show variation of healthcare utilisation patterns between primary and secondary healthcare services (page 4, lines 43-45) but that are not highlighted.

Response: Thank you—yes, this was part of our objective, and we did assess utilisation across both primary care and secondary care settings. Primary care utilisation was captured using primary care clinical encounters, prescriptions, and referrals recorded in CPRD, while secondary care utilisation was captured using hospital-based outcomes (hospitalisations/admissions) from linked HES data. We agree this contrast was not stated explicitly enough in the text. We have therefore (i) clarified the study objective and (ii) strengthened the Results narrative to highlight the differential patterns between primary and secondary care utilisation.

We revised the objective sentence to explicitly reference primary and secondary care between line 43-45 on page 4, as "…we aimed to quantify differences in healthcare utilisation **across primary care and secondary care services** in women with PNA compared with women with anxiety but not pregnant and women who were pregnant without anxiety."

We also added an explicit contrast when describing the postpartum periods, as follows "In Period-3 (73 days to 6 months postpartum), Period-4 (7–12 months postpartum) and Period-5 (13–24 months postpartum), primary care encounter, prescription, and referral rates remained higher for the PNA cohort than the matched cohorts, albeit the difference in rates were slightly lower than for period 2. **In contrast, secondary care utilisation (hospitalisations) was lower for the PNA cohort than both matched cohorts across Periods 3–5 (Supplemental Table 3).**"

3. Authors conducted sensitivity analyses which is very well justified. However, statement like, "Sensitivity analysis using complete cases only gave similar findings as the main analysis" without any data is very weak.

Response: Thank you. We agree that our original wording ("similar findings") was too general. We have now added quantitative complete-case results in the Results text and directed readers to Supplementary Table 5, which provides the full set of adjusted IRRs and ARDs for the complete-case analysis.

We revised the Results text to include an explicit numeric example (and cross-referenced Supplementary Table 5). Specifically, we added: "Complete-case analyses produced estimates consistent with the primary analysis (Supplementary Table 5). For example, in Period-2 (delivery to 72 days postpartum), the adjusted IRR for primary care clinical encounters comparing women with PNA with the anxiety-only cohort was **2.29 (95% CI 2.28 to 2.30)** (main analysis: **2.29 [2.28 to 2.29]**) and **2.32 (2.31 to 2.33)** compared with the pregnancy-only cohort (main analysis: **2.32 [2.31 to 2.32]**). Similar consistency was observed across outcomes and time windows."

Discussion and References

1. As mentioned there could be many more studies to show consistent results with the present study.

Response:Thank you. We agree that the Discussion should more clearly situate our findings within the broader evidence base on perinatal mental health and healthcare utilisation

(noting that much prior work focuses on perinatal depression or mixed perinatal mental health populations, and/or on early postnatal windows). We have therefore expanded the Discussion to cite additional relevant studies and to clarify how our work extends the literature by (i) focusing on a PNA-defined cohort, (ii) using two matched unexposed comparators, and (iii) reporting utilisation patterns across multiple postpartum windows up to 24 months.

We revised the first paragraph of the Discussion (**Page 12, Lines 44–58**) to read: "This study provides a comprehensive analysis of healthcare utilisation patterns in women with PNA compared to matched cohorts of women experiencing only anxiety or only pregnancy, using a large dataset of primary care records. Our findings show that women with PNA have consistently higher rates of primary care clinical encounters, prescriptions, and referrals to specialist care up to 24 months after delivery, indicating that healthcare demands of women with PNA are significantly higher. **These findings are consistent with evidence that perinatal mental health problems are associated with increased healthcare use and costs, although much of the existing literature has focused on perinatal depression or mixed perinatal mental health populations rather than anxiety-specific cohorts [4, 12, 13, 27]. UK primary care studies have also demonstrated marked changes in consultation patterns around childbirth, particularly in the early postnatal period, but have not provided matched comparisons focused on PNA.[18, 19]**"

2. References cited in page 12, Lines 42, 49, 59 are all wrong. Sometimes PMIDs are mentioned (page 12, Lines 24, 54). The manuscript needs thorough checking of the reference system.

Response: Thank you—this is correct. The two "PMID:" entries were placeholders that remained in the text due to a malfunction of the reference management (References) tab in Microsoft Word during formatting, which also contributed to mismatched in-text citation numbering in several places. We have now removed the PMIDs, replaced them with the correct **numbered references**, and rechecked the Discussion citation sequence to ensure all in-text citations align with the updated reference list.

Tables and Figures
There are minor errors with a suggestion that all of them should be self-explanatory. Footnotes could be used to explain abbreviated texts, and better resolution for figures are expected.

Response: Thank you. We agree and have revised the tables to ensure they are fully self-explanatory.

Reviewer: 2
Mr. Mashudu Rampilo, University of Johannesburg - Auckland Park Bunting Road Campus
Comments to the Author:
The paper examined differences in healthcare utilization among women with perinatal anxiety compared to two matched cohorts using electronic health records from CPRD.
I have included my specific comments on the document

Response: Thanks. Your comments placed in the document has been extracted as below and the point-to-point response was also placed as below.

Abstract

Page 3, line 19: You may indicate the total sample size, then do the breakdown by those with PNA, anxiety only and pregnancy only.

Response: Thank you. In the BMJ Open structured abstract format, the sample size is presented under *Participants* rather than *Setting*. The *Setting* line is intentionally limited to describing the data source and level of care, consistent with the journal's abstract structure; therefore, we have retained this formatting.

Page 3, line 21: Rather phrase it as records of 103,287 women

Page 3, line 23: Rather phrase it as records 3148 women

Response: Thank you. We agree the data source is electronic health records; however, the unit of inclusion and analysis is women, not "records." We therefore retain "women" to describe the cohorts, while clarifying that these women were identified from records in CPRD.

Revised text (Page 3, Participants):"Participants: The study included **records for** 103,287 women with PNA and two matched comparison cohorts: 413,148 women with anxiety but no pregnancy (anxiety-only cohort) and 413,148 women with pregnancy but no anxiety (pregnancy-only cohort). "

Page 3, line 23: Rephrase the subhead "Primary and secondary Outcome Measures" into "Data analysis"

Response: Thank you for the suggestion. We have retained the subheading "Primary and secondary outcome measures" because the structured abstract format is specified by the journal's author guidelines, and this heading is required to describe the planned outcomes rather than the analytical approach.

Page 3, line 51: rephrased the verb "exhibt" into "exhibited" and apply the same on other sections

Response: Thank you for this helpful suggestion. We have corrected "exhibt" to "exhibited" and thoroughly checked the manuscript to ensure consistent tense and grammar throughout.

STRENGTHS AND LIMITATIONS OF THIS STUDY

Page 4, line 6: rephrased the verb "utilises" into "utilized"

Response: Thank you for the suggestion. As this manuscript follows BMJ Open style and UK English conventions, we have retained the British spelling ("utilises") throughout for consistency.

Page 4, line 36: regarding the phrase "all studies", you could say most or majority of the studies, unless you have references for that statement

Response: Thank you for the suggestion. The term "all studies" has been replaced by "most studies" in the revised manuscript.

INTRODUCTION

Page 5, line 3: The introduction lacks the background data on the magnitude of PNA consequences, global, regional and local targets aligned with sustainable development goals and local policies

Response: Thank you. We agree the Introduction should cite more directly relevant evidence on healthcare utilisation around pregnancy and postpartum, and that the COVID-19-focused material was not essential to motivate the research question. We have therefore clarified the gaps we address and strengthened the supporting citations, while retaining the original structure of the paragraph. Specifically, we removed the COVID-19-focused reference(s) and replaced refs 7–10 with more relevant studies on (i) consultation patterns around childbirth in UK primary care and (ii) perinatal mental health service use/economic evidence.

We revised the paragraph in the Introduction (Page 4, Lines 38–52) as follows (new/changed text shown below):

"While previous research acknowledges increased healthcare demands among women with PNA, critical gaps remain in our understanding of the long-term trajectories of healthcare utilisation patterns **beyond the immediate postnatal period**. This includes a lack of detailed knowledge about how these patterns vary across primary and secondary healthcare services. **Although UK primary care studies have described marked changes in consultation patterns across pregnancy and the early postnatal period, with service use stabilising after the first months postpartum, these studies did not focus on women with PNA or provide matched comparisons.** Moreover, there is limited understanding of how women with PNA engage with healthcare services across different socio-demographic and ethnic groups, suggesting a need for more focused studies in this area."

We also replaced the original references 7–10 with the following (renumbered within the manuscript as refs 7–10):

7. Smith HC, Saxena S, Petersen I. Postnatal checks and primary care consultations in the year following childbirth: an observational cohort study of 309 573 women in the UK, 2006–2016. *BMJ Open* 2020;10:e036835.
8. Smith HC, Schartau P, Saxena S, Petersen I. The first 100 days after childbirth: cross-sectional study of maternal clinical events and health needs from primary care. *Br J Gen Pract* 2024;74:e580–e586.
9. Howard LM, Khalifeh H. Perinatal mental health: a review of progress and challenges. *World Psychiatry*2020;19:313–327.
10. Pisavadia K, Spencer LH, Tuersley L, Coates R, Ayers S, Edwards RT. Health economic evaluations of preventative care for perinatal anxiety and associated disorders: a rapid review. *BMJ Open* 2024;14:e068941.

Page 5, line 8: regarding the systematic review, Provide more details (such as place, time period and number of studies included) about the systematic review

Reponse Thank you. We agree that the description of the systematic review should be more informative. We have added a brief summary of the review characteristics (scope, time period covered, and number of included studies) at the point where it is first cited, while

keeping the text concise. We revised the sentence in the Introduction (Page 5, Line 8) to: “A systematic review **(including 26 studies, covering studies published from the inception of the database until April 2022 across global settings)** estimated the prevalence …”

Page 5, line 13, regarding the phrase “PNA”, Avoid starting a sentence with abbreviations

Response: Thank you. Although the abbreviation had been defined previously, we agree it is clearer stylistically not to begin a sentence with an abbreviation. We have revised the sentence to start with the full term while retaining “(PNA)” thereafter.

Page 5, line 19, regarding the place of reference, Align the full stops and references throughout the document

Response: Thank you. We agree. We have checked the manuscript throughout and standardised punctuation and reference placement as suggested.

Page 6, line 13, regarding the statement “Addressing this gap could enhanceour ability”, The study findings can be utilized beyond the study team as the aim is to publish the paper widely for knowledge sharing

Response: Thank you. We agree and have revised the wording to avoid implying relevance only to the study team.

Revised text in manuscript (Page 6, line 13): Changed from: “Addressing this gap could enhance **our** ability…” To: “Addressing this gap could enhance **healthcare systems’ and clinicians’** ability to identify and support women with PNA and to plan services across the perinatal and postpartum period.”

Page 6, line 31, rephrase the verb “seek” into “sought”

Response: Thank you. The word has been rephrase as suggested.

METHODS

Setting

Page 6, line 47, regarding the data setting statement “In the UK, primary care is typically the first point of contact for healthcare and acts as the ‘gatekeeper’ to the rest of the health service. Whilst a newly pregnant woman can refer themselves directly to a midwife service, they are likely to be seen in primary care for other health problems. Information about patients’ health is collected during consultations. ”. This can go to the introduction

Response: Thank you. We understand the suggestion; however, we have **not** moved or deleted this text. We consider it essential setting/context for interpreting healthcare utilisation outcomes derived from CPRD primary care records and linked HES data (ie, why primary care captures much of women’s healthcare use and how referrals to secondary care occur).

This information therefore fits the Setting subsection in Methods, consistent with BMJ Open reporting conventions. No change made.

Study population

Page 8, line 20, regarding the statement "Each eligible participant had to have at least two years of data", be clear about the data.

Response: Thank you for highlighting this. We have clarified that the "≥2 years of data" criterion refers to **continuous registration at their general practice** (with linkage to HES/IMD) for **at least 2 years before and 2 years after the index date**, as specified in the cohort definition.

Outcomes

Page 8, line 24, rephrase the subhead "Outcomes" into "Results"

Response: Thank you for the suggestion. We have not changed this subheading. "Outcomes" is a standard Methods subsection in BMJ Open–style reporting and is used to define the study endpoints and measurement windows prior to presenting findings in the Results section. We therefore retained the heading for consistency with the journal structure and to avoid confusing outcome definitions with results.

Page 8, line 31, regarding the phrase "the total number of referrals to special care". Did the study establish if the referrals were successful and how is this counted as a clinical encounter?

Response: Thank you for raising this point. In this study, "referrals" refer to referral records made in primary care (ie, the GP's decision/documentation to refer to specialist/secondary care). We did not assess whether referrals were subsequently attended, because referral completion is not consistently captured in CPRD and would require separate linkage/ascertainment. Referrals were counted as a distinct utilisation outcome (number of referrals) and were not counted as a "primary care clinical encounter." We have clarified this in the Methods (Outcomes) section.

Revised text in manuscript (Page 8, Lines 31–33):
"We measured referrals as the **number of primary care referral records to specialist/secondary care** recorded in CPRD. **Referral attendance was not assessed** and referrals were analysed as a **separate utilisation outcome**, not as a primary care clinical encounter."

Covariates

Page 9, line 8, regarding the phrase "2011-2015, and 2015-2020", correct it.

Response: Thank you for spotting this. We corrected the final index-year category to 2016–2020 to avoid overlap/duplication with the preceding period.

Page 9, line 8-13, regarding the statement "Geographical region covered North East, North West, Yorkshire and The Humber, East Midlands, West Midlands, East of England, London, South East, South West, and Northern Ireland," This should go under study setting

Response: Thank you for the suggestion. We agree that the *study setting* is already described in the "Setting/Data source" section (CPRD Aurum practices in England with linkage to HES&IMD). In contrast, geographical region is included here under Covariates because it was treated as an area-level confounder (and explored in subgroup/stratified analyses where applicable), rather than being used to describe the geographic coverage of the dataset. Therefore, we have retained this information in the Covariates section and made no change.

Statistical analysis
Page 9, line 40, regarding the subhead "Statistical analysis", Integrate outcomes, covariate and statistical analysis under one heading (Data Collection) and shorten the section.

Response: Thank you for the suggestion. We have retained the current subheadings ("Outcomes", "Covariates", and "Statistical analysis") because they follow standard reporting structure for observational studies (consistent with STROBE) and improve readability by separating *what was measured* from *how it was analysed*. Combining these under a single "Data Collection" heading would be less clear, as the current sections cover both definition/measurement **and** modelling/estimation, not only data collection.

Page 10, line 22, regarding the statement "All analyses were performed using Stata/MP 18.0",
Who performed the analysis?

Response: Thank you for this suggestion. We agree it is important to clarify who undertook the analyses. In line with BMJ Open reporting practice, author contributions are reported in the dedicated **"Contributors"** section, rather than within the Methods. We have therefore not amended the Methods text but have clarified in the Contributors section which author performed the statistical analyses.

Page 10, line 24, regarding the subhead "Patient and public involvement", Was this part of the study objectives? How many people were involved, how were they recruited, how was their qualitative data collected and analyzed. Where are the quotes, which tool was used for analysis.
Response: Thank you for this comment. The "Patient and public involvement" section is a BMJ-required reporting item describing how PPI contributors informed the study's design and dissemination; it is not a qualitative substudy and was not part of the study objectives. Our Patient Advisory and Clinical Advisory Groups provided feedback on study objectives, relevance, comparisons between cohorts, and communication of findings. No interviews/focus groups were conducted for research purposes, no qualitative dataset was generated, and therefore no quotations or qualitative analytic tools apply. We have clarified this in the PPI section by adding the statement as "**PPI input was advisory and did not involve collection of qualitative research data (eg, interviews), therefore no quotations or qualitative analytic tools apply.**".

Page 10, line 34, regarding the word "manuscript" in the sentence "including the database study reported in this manuscript", This status will change into a paper.

Response: We have used "paper" to replace "manuscript" as suggested.

RESULTS

Page 1o, line 51, regarding the statement “In total, 103,287 women with PNA, 413,148 women with anxiety but no pregnancy, and 413,148 pregnant women but no anxiety were included in the study. ” This should is already covered under study participants

Response: This comment appears to be based on a misunderstanding. The cohort sizes are not reported in the Methods section under Study population; they are reported in the Abstract under *Participants*, as required for a structured abstract. The sentence on Page 10, line 51 is the first presentation of the final analytic sample size in the main text Results, which is standard and helps orient readers.

DISCUSSION

Page 13, line 59, regarding the phrase “perinatal anxiety”, Abbreviated already, please check other abbreviations as well & Page 15, line 13, regarding the phrase “perinatal anxiety”, Abbreviated already. Same comment
Response: We have addressed this. In the locations highlighted (Page 13, line 59; Page 15, line 13), we replaced “perinatal anxiety” with the abbreviation PNA as suggested.

Page 15, line 26, regarding the sentence “This study has notable strength”, You have this section on page 9 & Page 16, line 10, regarding the statement “There are several limitations that should be acknowledged.” You have this on page 3

Response: Thank you for raising this. We note that BMJ Open requires a separate “Strengths and limitations of this study” box (present earlier in the manuscript). In addition, we retain a brief strengths/limitations summary within the Discussion to support interpretation of the findings in the main text. These two components serve complementary purposes (a structured box for rapid appraisal vs a narrative discussion linked to interpretation), and therefore we have not removed the Discussion statement.

CONCLUSIONS
Page 16, line 45, regarding the subhead “CONCLUSIONS”, Align with the conclusions in the abstract section. Think about the research question

Response: We note this comment. The Conclusions section in the main text already directly reflects the study objective/research question (differences in healthcare utilisation in women with PNA compared with matched anxiety-only and pregnancy-only cohorts) and is consistent with the abstract conclusion. The abstract provides a concise summary, while the main-text Conclusions reiterate the same message in the context of the full results and interpretation. Therefore, we have not made changes to the Conclusions subheading or content.

Page 16, line 52, regarding the phrase “perinatal anxiety”, Abbreviated already &
Page 16, line 56, regarding the phrase “perinatal anxiety”, Abbreviated already
Response: We have replaced “perinatal anxiety” with the abbreviation PNA as suggested.

ACKNOWLEDGEMENTS
Page 17, line 17, regarding the subhead “ACKNOWLEDGEMENTS”, Only acknowledge the contributions from different individuals or organizations, etc. Do not include data collection
Response: Thank you for the suggestion. The CPRD and HES statements included under Acknowledgements are mandatory wording required by CPRD/HES data governance and licensing terms for all publications using these datasets. They function as a required data-source acknowledgement/disclaimer (rather than describing “data collection” by the

study team) and therefore need to remain in the manuscript to ensure compliance with the data provider requirements.

Page 17, line 39, about the word “and”, Different font.

Response: The font has been unified.

Page 18, line 17, regardinig the “PATIENT AND PUBLIC INVOLVEMENT” , This section is also on page 9
Response: Thank you for noting this. The Patient and Public Involvement (PPI) information appears in two places for different purposes: (1) within the Methods to describe how PPI informed the study design and interpretation, and (2) as a separate ‘Patient and public involvement’ statement in the end-matter, which is required by BMJ Open for transparency and indexing. The text is therefore intentionally presented in both locations to meet journal reporting requirements. We have checked for consistency between the two locations.

REFERENCES
Page 19, line 12, regarding the subhead “REFERENCES”, Align like other headings

Response: Thank you. We have aligned the REFERENCES heading to match the formatting used for other main headings throughout the manuscript.

Reviewer: 3
Dr. Yaxu Zhuang , University of Colorado Denver Anschutz Medical Campus
Comments to the Author:
The manuscript is generally well written and clearly presented. However, there is a major concern regarding the statistical methodology used for the analysis of longitudinal outcomes. The authors estimated outcome rates within each follow-up period using Poisson regression models and evaluated associations between PNA and outcomes using conditional Poisson regression, both unadjusted and adjusted for ethnicity, geographic region, deprivation, comorbidity, obesity, and smoking status.

Response: Thank you for summarising our analytical approach. We confirm that we estimated period-specific absolute utilisation rates and period-specific associations (IRRs and ARDs) using Poisson models, with conditional Poisson regression aligned to our matched cohort design. We have clarified in the Statistical analysis section that our estimations are population-level healthcare utilisation rates by pre-specified, non-overlapping perinatal periods, rather than modelling individual trajectories across time.

We added to Statistical analysis: “**Our analyses focused on population-level healthcare utilisation (rates of events per person-time) within pre-defined perinatal periods, rather than individual-level longitudinal trajectory modelling.**”

Given that each individual contributes repeated measurements across multiple follow-up periods, the data are inherently longitudinal and correlated. As such, the use of a generalized linear mixed model (GLMM), with appropriate random effects to account for within-subject correlation, would be more statistically appropriate than fitting separate Poisson models for each period.

Response: We appreciate this point. Although each woman contributes observations across multiple follow-up windows, our analysis was designed to estimate period-specific utilisation rates and period-specific rate ratios at the population level using non-overlapping time windows (Period 1–5), which is standard for rate-based health service utilisation analyses. Our primary aim was not to model within-person correlation or trajectories, but to quantify utilisation rates and contrasts within each clinically meaningful perinatal period under a matched cohort design. We have therefore retained the period-specific modelling approach, and we now explicitly justify this choice and the interpretation of the estimates in the Statistical analysis section.

Moreover, while the authors state that the longitudinal nature of the data allows examination of period effects and how the association between PNA and healthcare outcomes evolves over time, this claim is not adequately supported by the current modeling strategy. A GLMM framework with interaction terms between time period and PNA would more rigorously demonstrate these longitudinal and period-specific effects and provide a stronger basis for the authors' conclusions.

Response: Thank you — we agree the wording should accurately reflect what our modelling strategy supports. Our intention was to describe how utilisation contrasts differ across predefined postpartum/pregnancy windows (Period 1–5), rather than to claim formal inference on a continuous longitudinal trajectory. We have therefore revised the manuscript wording to make this explicit: our period-specific estimates provide a clear comparison of utilisation across clinically meaningful time windows, and our "period effects" refer to (i) differences across perinatal periods and (ii) calendar index-year analyses assessing temporal variation. We have updated the Statistical analysis text to clarify these points and avoid overstatement as "**By partitioning follow-up into five predefined perinatal periods, we compared period-specific utilisation rates and associations to describe how differences between cohorts vary across pregnancy and postpartum windows.**"

VERSION 2 - REVIEW

**Reviewer**	**3**
**Name**	**Zhuang , Yaxu**
**Affiliation**	**University of Colorado Denver Anschutz Medical Campus, Department of Medicine**
**Date**	**24-Feb-2026**
**COI**	

The manuscript is well written and addresses an important topic; however, there are two major methodological issues that should be addressed before the manuscript is suitable for publication.

First, the study defines five perinatal time periods: (1) pregnancy to delivery, (2) delivery to 72 days postpartum, (3) 73 days to 6 months postpartum, (4) 7–12 months postpartum, and (5) 13–24 months postpartum. The authors state that the analysis focuses on population-level healthcare utilization (rates of events per person-time) rather than individual-level longitudinal trajectories. The issue is, individuals contribute data across multiple periods, the observations are inherently correlated within person. If the goal is population-averaged inference, a generalized estimating equations (GEE) approach should be considered to account for within-person correlation across periods. Alternatively, if the objective is to model individual-level variability, a generalized linear mixed model (GLMM) with subject-level random effects would be more appropriate. The current justification—that conclusions are not dependent on modelling within-person random effects because periods are non-overlapping—does not fully address the potential correlation introduced by repeated contributions from the same individuals over time. Clarification and potential re-specification of the modeling framework are warranted.

Second, the set of covariates included in the analysis appears limited, given the scale and richness of electronic health record (EHR) data. The authors adjust for age, index year, geographical region, deprivation, ethnicity, smoking status, body mass index, and comorbidities; however, EHR-based studies typically allow for more comprehensive adjustment, including healthcare access factors, prior utilization patterns, medication use, obstetric history, and other clinical or social determinants. A more systematic variable selection strategy (e.g., theory-driven selection, penalized regression, or high-dimensional propensity approaches) would help identify key predictors and reduce residual confounding. Expanding the covariate set would strengthen the validity and interpretability of the findings, particularly given that the primary outcome—healthcare utilization measured by the number of primary care encounters—is likely influenced by multiple structural and clinical factors.

VERSION 2 - AUTHOR RESPONSE

Reviewer: 3
Dr. Yaxu Zhuang , University of Colorado Denver Anschutz Medical Campus
Comments to the Author:
The manuscript is well written and addresses an important topic; however, there are two major methodological issues that should be addressed before the manuscript is suitable for publication.

First, the study defines five perinatal time periods: (1) pregnancy to delivery, (2) delivery to 72 days postpartum, (3) 73 days to 6 months postpartum, (4) 7–12 months postpartum, and (5) 13–24 months postpartum. The authors state that the analysis focuses on population-level healthcare utilization (rates of events per person-time) rather than individual-level longitudinal trajectories. The issue is, individuals contribute data across multiple periods, the observations are inherently correlated within person. If the

goal is population-averaged inference, a generalized estimating equations (GEE) approach should be considered to account for within-person correlation across periods. Alternatively, if the objective is to model individual-level variability, a generalized linear mixed model (GLMM) with subject-level random effects would be more appropriate. The current justification—that conclusions are not dependent on modelling within-person random effects because periods are non-overlapping—does not fully address the potential correlation introduced by repeated contributions from the same individuals over time. Clarification and potential re-specification of the modeling framework are warranted.

Response: We thank the reviewer for raising this. Women can contribute observations across multiple non-overlapping perinatal periods (and, for some women, across multiple pregnancies), so within-person correlation may arise. Our estimation is population-averaged period-specific healthcare utilisation rates rather than individual trajectory parameters; therefore we did not pursue a subject-specific random-effects GLMM as the primary approach. Instead, we have now restructured the dataset into long format and refitted the analysis using two approaches that account for within-person correlation: (i) Poisson rate models with robust (sandwich) standard errors clustered at the woman level, and (ii) population-averaged Poisson GEE models with an exchangeable working correlation and robust variance. Period-specific incidence rate ratios were derived from cohort-by-period interaction terms. Results from both approaches were consistent with the original conclusions; GEE estimates were slightly closer to the null but the direction and time-pattern of effects were unchanged. We have updated the Methods to describe these sensitivity analyses and added the results in Supplementary Table 6 (as below).

Supplemental Table-6. Sensitivity analyses for the primary outcome: period-specific primary care clinical encounters rate ratios comparing cohorts, using clustered Poisson and population-averaged GEE Poisson models.

	**PNA vs matched cohort 1 (Anxiety without pregnancy)**<br><br>**IRR (clustered Poisson), 95% CI**	**PNA vs matched cohort 1 (Anxiety without pregnancy)**<br><br>**IRR (GEE), 95% CI**	**PNA vs matched cohort 2 (Pregnancy without anxiety)**<br><br>**IRR (clustered Poisson), 95% CI**	**PNA vs matched cohort 2 (Pregnancy without anxiety)**<br><br>**IRR (GEE), 95% CI**
**Period-1: Index date-delivery date**	1.766 (1.756, 1.775)	1.685 (1.676, 1.694)	1.641 (1.633, 1.648)	1.532 (1.524, 1.539)
**Period-2: delivery date-72 days postpartum**	2.290 (2.276, 2.304)	2.150 (2.136, 2.164)	2.285 (2.273, 2.297)	2.027 (2.015, 2.039)
**Period-3: 73 days-6 months postpartum**	1.277 (1.268, 1.286)	1.175 (1.167, 1.184)	1.390 (1.381, 1.399)	1.223 (1.215, 1.232)
**Period-4: 7-12 months postpartum**	1.248 (1.240, 1.257)	1.164 (1.156, 1.171)	1.516 (1.507, 1.526)	1.368 (1.359, 1.376)
**Period-5: 13-24 months postpartum**	1.249 (1.241, 1.257)	1.182 (1.175, 1.189)	1.558 (1.548, 1.567)	1.444 (1.435, 1.453)

IRRs compare primary care clinical encounter rates in the perinatal anxiety (PNA) cohort versus each matched comparator cohort within the same time period.

Clustered Poisson models used robust (sandwich) standard errors clustered at the woman level to account for within-person correlation across repeated period contributions (and multiple pregnancies where applicable).

Population-averaged Poisson GEE models used a log link, an exchangeable working correlation structure, and robust variance estimates.

All models included a log(person-time) offset (person-time in years) and were adjusted for ethnicity, deprivation decile, alcohol use, smoking status, obesity, and Charlson Comorbidity Index, consistent with the main analysis.

Period-specific IRRs were obtained from cohort-by-period interaction terms.

IRR>1 indicates higher encounter rates in the PNA cohort than in the matched comparator cohort for that period.

We have ensured all changes described above are now reflected in the revised manuscript:

In the methods section between line 18 on page 10 and line 1 on page 11, we added "*Because women contribute observations across multiple perinatal periods (and some contribute more than one pregnancy episode), we conducted sensitivity analyses to account for within-person correlation across repeated period-specific observations. We restructured the dataset into long format with one record per woman–episode–period and refitted models using (i) Poisson regression with robust standard errors clustered at the woman level and (ii) population-averaged Poisson GEE models (log link, Poisson family) with an exchangeable working correlation and robust variance. Period-specific incidence rate ratios were derived from cohort-by-period interaction terms.*"

In the Results section between line 3-7 on page 14, we added "*Sensitivity analyses that accounted for within-person correlation using woman-clustered robust Poisson models and population-averaged Poisson GEE models produced consistent period-specific estimates for consultation rates; GEE estimates were slightly attenuated towards the null, but the direction and overall time-pattern of differences between cohorts were unchanged (Supplementary Table 6)*."

Second, the set of covariates included in the analysis appears limited, given the scale and richness of electronic health record (EHR) data. The authors adjust for age, index year, geographical region, deprivation, ethnicity, smoking status, body mass index, and comorbidities; however, EHR-based studies typically allow for more comprehensive adjustment, including healthcare access factors, prior utilization patterns, medication use, obstetric history, and other clinical or social determinants. A more systematic variable selection strategy (e.g., theory-driven selection, penalized regression, or high-dimensional propensity approaches) would help identify key predictors and reduce residual confounding. Expanding the covariate set would strengthen the validity and interpretability of the findings, particularly given that the primary outcome—healthcare utilization measured by the number of primary care encounters—is likely influenced by multiple structural and clinical factors.

Response: Thank you for this thoughtful suggestion. We agree that primary care utilisation is influenced by a wide range of clinical, behavioural, and structural determinants, and that richer adjustment can strengthen interpretability in EHR-based analyses. In this study, covariates were selected a priori based on clinical/epidemiological rationale and on what was consistently available and likely to be comprehensively recorded in the linked CPRD Aurum resources used (including age, index year, region, deprivation, ethnicity, smoking, obesity/body mass index, and comorbidity burden). Several domains suggested by the reviewer—such as detailed

obstetric history recorded in maternity systems, more granular healthcare access measures, and comprehensive medication use/prior utilisation patterns—were not available in the linked extract underpinning this analysis. In addition, the original patient-level CPRD extract used for the study is no longer accessible to us under CPRD governance requirements and the addition of extra covariates would also require a major amendment to the approved protocol. We are therefore unable to undertake additional bespoke data extraction or derive new covariates at this stage.

We have revised the manuscript to more explicitly acknowledge these constraints and the potential for residual confounding due to unmeasured determinants of utilisation. We also clarify that the primary objective is a descriptive comparison of population-level utilisation rates between cohorts across clinically defined perinatal periods rather than causal estimation of an intervention effect; nonetheless, unmeasured structural and clinical factors may contribute to the observed differences.

We strengthened the Discussion (Limitations) to acknowledge residual confounding from determinants not captured in the linked extract (page 18, lines 14-20). The added text reads: "*Residual confounding may remain because some important determinants of healthcare utilisation (e.g., detailed obstetric history captured in maternity systems, healthcare access factors, prior utilisation patterns, and more granular medication use) were not available within the linked datasets used in this study. Future studies with access to richer data sources should consider incorporating these domains and using more systematic covariate selection strategies to further strengthen inference.*"
